# Selective Expression of Osteopontin in ALS-resistant Motor Neurons is a Critical Determinant of Late Phase Neurodegeneration Mediated by Matrix Metalloproteinase-9

**DOI:** 10.1038/srep27354

**Published:** 2016-06-06

**Authors:** Yuta Morisaki, Mamiko Niikura, Mizuho Watanabe, Kosuke Onishi, Shogo Tanabe, Yasuhiro Moriwaki, Takashi Okuda, Shinji Ohara, Shigeo Murayama, Masaki Takao, Sae Uchida, Koji Yamanaka, Hidemi Misawa

**Affiliations:** 1 Division of Pharmacology, Faculty of Pharmacy, Keio University, Tokyo 105-8512, Japan; 2 Department of Neurology, Matsumoto Medical Center, Chushin-Matsumoto Hospital, Matsumoto 399-0021, Japan; 3 Department of Neuropathology, Tokyo Metropolitan Institute of Gerontology, Tokyo 173-0015, Japan; 4 Department of Autonomic Neuroscience, Tokyo Metropolitan Institute of Gerontology, Tokyo 173-0015, Japan; 5 Department of Neuroscience and Pathobiology, Research Institute of Environmental Medicine, Nagoya University, Nagoya 464-8601, Japan

## Abstract

Differential vulnerability among motor neuron (MN) subtypes is a fundamental feature of amyotrophic lateral sclerosis (ALS): fast-fatigable (FF) MNs are more vulnerable than fast fatigue-resistant (FR) or slow (S) MNs. The reason for this selective vulnerability remains enigmatic. We report here that the extracellular matrix (ECM) protein osteopontin (OPN) is selectively expressed by FR and S MNs and ALS-resistant motor pools, whereas matrix metalloproteinase-9 (MMP-9) is selectively expressed by FF MNs. OPN is secreted and accumulated as extracellular granules in ECM in three ALS mouse models and a human ALS patient. In SOD1^G93A^ mice, OPN/MMP-9 double positivity marks remodeled FR and S MNs destined to compensate for lost FF MNs before ultimately dying. Genetic ablation of OPN in SOD1^G93A^ mice delayed disease onset but then accelerated disease progression. OPN induced MMP-9 up-regulation via αvβ3 integrin in ChAT-expressing Neuro2a cells, and also induced CD44-mediated astrocyte migration and microglial phagocytosis in a non-cell-autonomous manner. Our results demonstrate that OPN expressed by FR/S MNs is involved in the second-wave neurodegeneration by up-regulating MMP-9 through αvβ3 integrin in the mouse model of ALS. The differences in OPN/MMP-9 expression profiles in MN subsets partially explain the selective MN vulnerability in ALS.

Amyotrophic lateral sclerosis (ALS) is an adult-onset, fatal neurodegenerative disease characterized by progressive degeneration of motor neurons (MNs) and skeletal muscle denervation and atrophy. However, strong evidence suggests the MN pools involved in eye movement and pelvic sphincter control are largely spared in ALS^1^,^2^. Moreover, within a given MN pool, differences in vulnerability between MN subtypes (e.g. alpha/gamma or fast/slow) are a fundamental but unexplained feature of ALS. α-MNs are divided into three subtypes: fast-twitch fatigable (FF), fast-twitch fatigue-resistant (FR) and slow-twitch fatigue-resistant (S). The large-diameter low-excitability FF MNs innervate type IIB muscle fibers and degenerate early in the ALS disease course, while medium-excitability FR MNs innervating type IIA muscle fibers follow after that. The high-excitability S MNs innervating type I muscle fibers are largely resistant to degeneration and are preserved, even late in the disease course. Although molecular markers for MNs have been available for several years^3^,^4^,^5^,^6^, the absence of markers that distinguish FF from FR MNs has hindered analysis of the early selective degeneration of MN subtypes.

The majority of ALS cases are sporadic, but about 10% are inherited, and dominant mutations in the gene for copper/zinc superoxide dismutase (SOD1) are the frequent cause of inherited ALS^7^. Transgenic rodent models of SOD1-linked ALS recapitulate the disease course with pool and subtype selectivity akin to those seen in ALS patients^8^. In most ALS patients, the first neurological symptoms occur during the fifth or sixth decade of life, after which years of subclinical disease progression are followed by relentlessly devastating clinical stages. Detailed longitudinal analyses of rodent ALS models have shown that a variety of physiological or biological disease-associated changes occur long before the onset of clinical disease^1^,^9^,^10^. For example, ALS models and patients both exhibit substantial axonal retraction and muscle denervation during the protracted presymptomatic phase. These early neuromuscular abnormalities, which involve FF motor axons and occur around P50 or earlier in SOD1^G93A^ model^11^,^12^, are compensated for by adaptive axonal sprouting and re-innervation by FR and S MNs^13^. However, this early protective effect begins to fail around the time of clinical onset, when the spreading neurodegeneration affects not only FF but also FR MNs^12^,^14^. In SOD1^G93A^ mice, overt signs of clinical muscle paralysis are first detected around P100. At the same time, neuroinflammation involving astrogliosis and microgliosis becomes evident^1^. The activated astrocytes and microglia have a non-cell-autonomous influence on ALS progression, exerting both deleterious and protective effects^15^,^16^.

Our hypothesis is that unknown signals which are expressed in MNs and released from them, along with mutant SOD1 or misfolded wild-type SOD1 species^17^,^18^,^19^,^20^,^21^, serve as local triggers and govern focal spread of ALS disease propagation. One possibility is that the secreted signal is an extracellular matrix (ECM) component, for example osteopontin (OPN), which is a widely expressed pleiotropic protein involved in a broad array of cell functions, including bone remodeling, wound healing, cancer metastasis, vascularization, the response to ischemia, immune responses and inflammation^22^,^23^. We recently observed that OPN is strongly and selectively expressed by α-MNs in the mouse spinal cord, and that it can serve as a marker distinguishing α- from γ-MNs^24^. It has also been reported that OPN is a potential ALS-specific cerebrospinal fluid biomarker^25^,^26^. In addition, OPN enhances matrix metalloproteinase-9 (MMP-9) transcription^27^,^28^,^29^ and activation^30^ through αvβ3 integrin-mediated signal transduction^30^,^31^. This is noteworthy, as MMP-9 is exclusively expressed by fast MNs and selectively conditions them to become vulnerable to mutant SOD1-induced toxicity^32^.

In the present study, we examined OPN’s ability to serve as a marker of FR/S MN subtypes and its role in mediating the selective vulnerability of MNs in ALS mouse models. Our results suggest OPN is a MN type-specific factor involved in the second-wave of MN degeneration mediated by MMP-9, and that OPN also modulates neuronal-glial interaction during the chronic inflammation seen in ALS mice.

## Results

### OPN is selectively expressed by FR/S MNs

**Figure 1:**
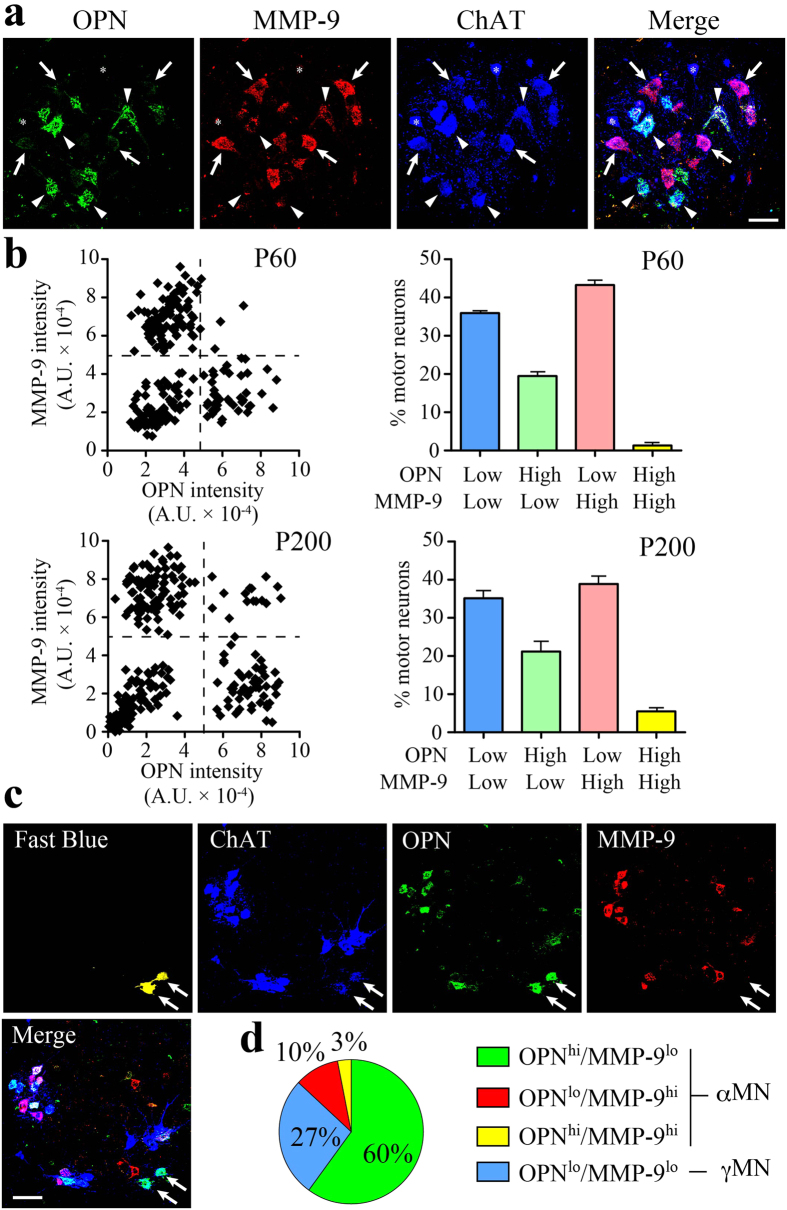
Contrasting localization of OPN and MMP-9 in mouse spinal motor neurons. (**a**) Immunostaining of OPN (green) is segregated from that of MMP-9 (red) in ChAT (blue)-positive MNs in the mouse lumbar spinal cord on P200. Arrows, OPN^lo^/MMP-9^hi^ MNs; arrowheads, OPN^hi^/MMP-9^lo^ MNs; asterisks, OPN^lo^/MMP-9^lo^ MNs. (**b**) Scatter and bar diagrams of OPN and MMP-9 fluorescence intensity in each MN (see Experimental Procedure). The scatter diagrams are representative data from P60 (n = 231 MNs) or P200 (n = 189 MNs) mouse. The bar diagrams show the percentage of MNs present in each quadrant (n = 3, with 363-595 MNs/10–12 histological sections analyzed per experimental animal). (**c**) Retrograde labeling of ALS-resistant MNs (arrows, false-colored yellow) in the lower lumbar spinal cord (L6) after fast blue injection into the external anal sphincter muscle. Immunostaining for ChAT (blue), OPN (green) and MMP-9 (red). (**d**) Fast blue-labeled MN fractions in the four groups defined by their OPN/MMP-9 expression profiles (with 51 MNs analyzed collectively from 6 experimental animals). Scale bar, 50 μm (**a**) and 100 μm (**c**). See also Supplemental Fig. 1.

The lack of histological markers with which to distinguish among MN subtypes (FF, FR and S) has precluded detailed pathological analyses in rodent ALS models. To gain mechanistic insight into the subtype-specific MN degeneration characteristic of ALS, we performed immunohistochemical (IHC) analyses in the mouse spinal cord by triple staining for OPN, MMP-9 and choline acetyltransferase (ChAT), an established MN marker. We noticed that OPN and MMP-9 were clearly segregated among ChAT-positive MNs (Fig. 1a), which could be classified into four groups based on their OPN and MMP-9 fluorescence intensities (Fig. 1b). On P60, the OPN-low/MMP-9-low MN fraction was 35%, the OPN-high/MMP-9-low fraction was 20%, the OPN-low/MMP-9-high fraction was 43%, and OPN-high/MMP-9-high MNs were barely detectable (1%). On P200, the respective MN fractions were nearly the same as on P60, except that the OPN-high/MMP-9-high fraction had increased to 6%. Importantly, the OPN-low/MMP-9-low MN fraction was consistent with the reported proportion of γ-MNs in the mouse spinal cord^1^,^3^.

In ALS, oculomotor neurons and Onuf’s nuclei are resistant in degeneration. We therefore injected a fluorescent tracer, fast blue, into the external anal sphincter muscle to label the mouse equivalent of Onuf’s nucleus and then classified the retrogradely labeled MNs based on their staining for OPN, MMP-9 and ChAT. The majority of fast blue-labeled, ChAT-positive MNs were OPN-high/MMP-9-low (Fig. 1c,d); among α-MNs, the OPN-high/MMP-9-low MN fraction was 82% (calculated excluding γMNs). Similarly, we detected virtually no MMP-9-positive MNs in the ALS-resistant oculomotor nucleus (n = 3, with 120–205 MNs analyzed per experimental animal), though OPN expression was detectable in many MNs (Supplementary Fig. 1a). These results suggest OPN is expressed in ALS-resistant MNs, but MMP-9 is not.

We previously detected OPN in MN perikarya and at neuromuscular junctions (NMJs)^24^. To determine which motor units express OPN, NMJs in the medial gastrocnemius muscle, which has mixed fast and slow muscle fibers, were stained with goat anti-OPN antibody, fluorescently labeled α-bungarotoxin (α-BGTX), and mouse monoclonal antibodies against myosin heavy chain (MyHC) isoform I, IIA or IIB. OPN-positive MN terminals were detected on type I and type IIA fibers, but not on type IIB fibers (Supplementary Fig. 1b), suggesting OPN is expressed in FR and S MNs. It thus appears that the ALS-resistant OPN-high/MMP-9-low MNs are FR and S type, OPN-low/MMP-9-high MNs are FF type, and OPN-low/MMP-9-low MNs are γ-MNs. The nature of the OPN-high/MMP-9-high MNs remains unknown.

### OPN/MMP-9 double positive MNs appear in ALS model mice during disease progression

**Figure 2:**
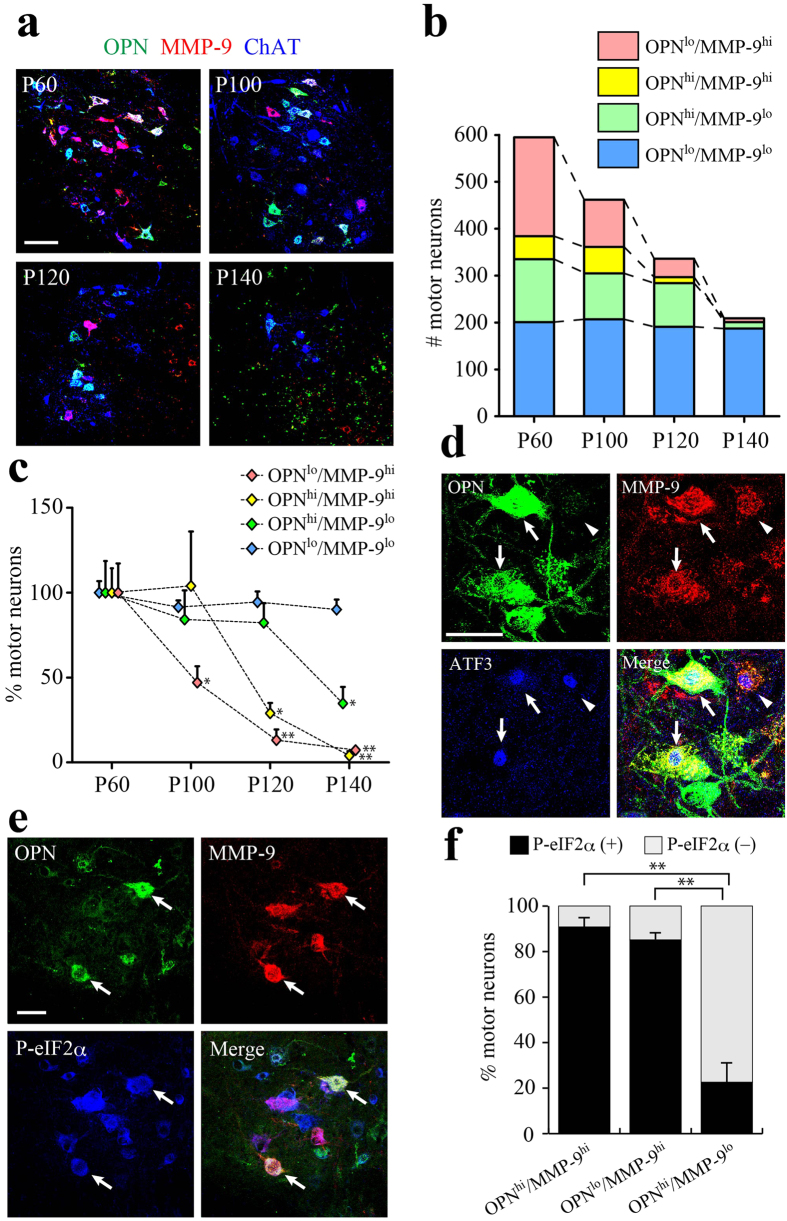
Remodeled FR/S motor neurons express MMP-9 and become vulnerable to mutant SOD1 toxicity. (**a**) Immunostaining for OPN (green), MMP-9 (red) and ChAT (blue) in the lumbar spinal cord of SOD1^G93A^ mice at the indicated disease stages (P60, P100, P120 and P140). (**b**) Changes in the numbers of MNs classified based on their OPN/MMP-9 expression profile at each stage (12 sections/mice, see Experimental Procedures). (**c**) Quantitative analysis of the number of MNs classified based on their OPN/MMP-9 expression profile. The each number of MNs detected at P60 were set to 100% (n = 3–4; *p < 0.05, **p < 0.01, compared with P60 values in each group, one-way ANOVA with Tukey-Kramer post hoc tests). (**d**) Immunostaining for OPN (green), MMP-9 (red) and ATF3 (blue) in the lumbar spinal cord of SOD1^G93A^ mice on P60. Arrows indicate OPN/MMP-9/ATF3 triple positive cells; arrowheads indicate MMP-9/ATF3 double positive cells. (**e**) Immunostaining for OPN (green), MMP-9 (red) and P-eIF2α (blue) in the lumbar spinal cord of SOD1^G93A^ mice on P60. Arrows indicate OPN/MMP-9/P-eIF2α triple positive cells. (**f**) P-eIF2α-positive cell fractions among the indicated MN types classified based on their OPN/MMP-9 expression profile in the lumbar spinal cord of SOD1^G93A^ mice on P60 (n = 3; **p < 0.01, one-way ANOVA with Tukey-Kramer post hoc tests). Scale bar, 100 μm (**a**) and 50 μm (**d**, **e**). See also Supplemental Fig. 2.

We next used immunohistochemistry to analyze the changes in OPN/MMP-9 expression profiles among MNs during disease progression in SOD1^G93A^ mice (Fig. 2a–c). The overall number of ChAT-positive MNs gradually declined as the disease progressed (Fig. 2b). On P60, although the fraction of each MN type was nearly the same as in WT mice at the same age (Fig. 1b), a small but significant increase in the OPN-high/MMP-9-high fraction (9%) was observed (Supplementary Fig. 2a), as compared to P60 WT MNs. And by P100 the OPN-high/MMP-9-high fraction reached 12% in SOD1^G93A^ mice (Supplementary Fig. 2a). In addition, as shown statistically in Fig. 2c, the OPN-low/MMP-9-high MN fraction (FF) began to decline before disease onset (P100) and the reduction continued throughout the disease course. By contrast, decreases in the OPN-high/MMP-9-high MN fraction occurred between P100 and P120 and in the OPN-high/MMP-9-low MN fraction between P120 and P140. Presumptive γ-MNs (OPN-low/MMP-9-low) were preserved even on P140. Supplementary Fig. 2c contains representative zymography and Western blots showing increased MMP-9 expression before (P60) or around (P100) the clinical disease onset in the spinal cord of SOD1^G93A^ mice. We also analyzed OPN/MMP-9 expression profile of MNs in transgenic mice expressing human wild-type SOD1 (WT-hSOD1) (Supplementary Fig. 2b) and found nearly the same population observed in non-transgenic WT mice (Fig. 1b), suggesting the changes in OPN/MMP-9 expression profile are driven by SOD1^G93A^ but not by transgenic expression of WT-hSOD1.

Activation of the unfolded protein response (UPR) and ER stress is one of the earliest molecular events detected in SOD1^G93A^ MNs^33^ and in patients with familial or sporadic ALS (FALS or SALS, respectively)^34^. Two ER stress markers, ATF3 (Fig. 2d) and phosphorylated eIF-2α (P-eIF2α) (Fig. 2e), were detected on P60 in OPN-high/MMP-9-high and OPN-low/MMP-9-high (FF) SOD1^G93A^ MNs. When quantified, the P-eIF2α-positive fraction on P60 was more than 90% among OPN-high/MMP-9-high MNs and 85% among OPN-low/MMP-9-high MNs (Fig. 2f). On the other hand, only 20% of OPN-high/MMP-9-low (FR/S) MNs were positive for the ER stress marker (Fig. 2f). Consistent with the finding by Kaplan *et al.*^32^, we detected no P-eIF2α-expressing MNs in WT mice (P200). We hypothesized that OPN-high/MMP-9-high MNs are “remodeled” FR or S MNs that compensated for the early loss of the highly vulnerable FF motor units. To explore that possibility, we analyzed NMJs in the medial gastrocnemius muscle of SOD1^G93A^ mice on P100. OPN-positive MN terminals were clearly detectable at NMJs on type IIB muscle fibers (Supplementary Fig. 2d upper panels). Furthermore, the areas innervated by OPN-positive MN terminals had expanded on hypertrophic muscle fibers, but had disappeared from atrophic fibers (Supplementary Fig. 2d lower panels). Sciatic nerve axotomy in normal adult mice did not change the relative proportions of MN subtypes grouped based on their OPN/MMP-9 expression profiles (data not shown), which indicates it is not the case that FF MNs express OPN upon denervation. Instead, these results indicate that OPN-high/MMP-9-high MNs are remodeled FR or S MNs innervating MN terminal areas vacated due to early loss of vulnerable FF MNs. The similar preservation of MMP-9-positive MNs was also observed in SOD1^G85R^ mice after loss of FF MNs^35^.

### Accumulation of OPN-positive granular deposits in the spinal cords of ALS mice during disease progression and in a mutant SOD1-linked FALS patient

**Figure 3:**
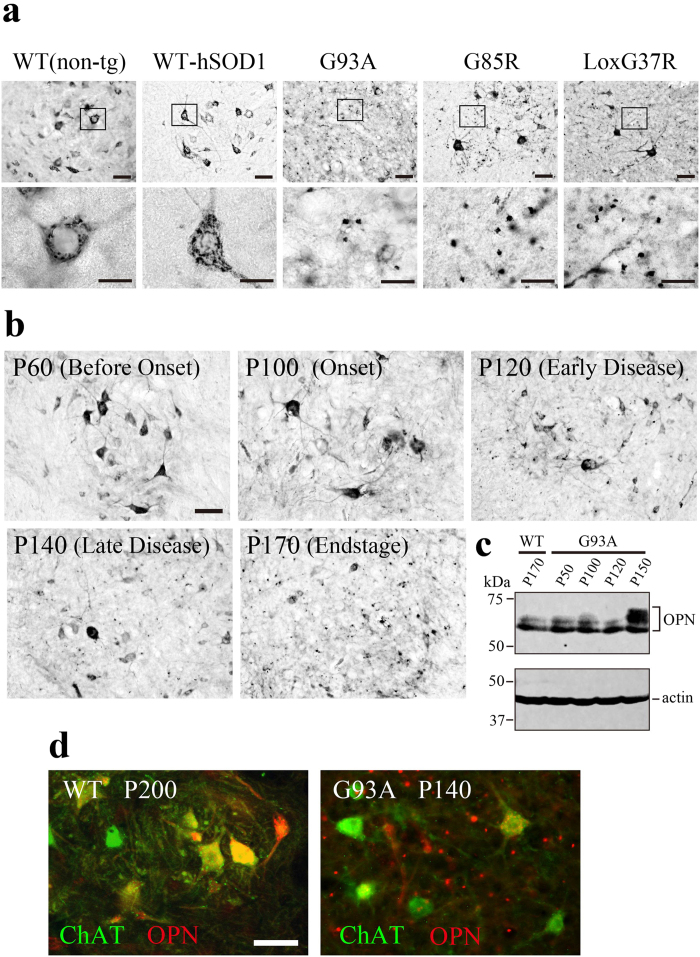
Accumulation of extracellular OPN-immunopositive granules in the spinal cords of ALS model mice during disease progression. (**a**) OPN immunostaining in lumbar spinal cord sections from SOD1^G93A^ (G93A), SOD1^G85R^ (G85R) and LoxSOD1^G37R^ (LoxG37R) mutant mice at their respective end stages, or from non-transgenic control mice (WT) at P200 or human wild-type SOD1-transgenic mice (WT-hSOD1) at P180. OPN signals were detected within MN cell bodies in the WT and WT-hSOD1 mice. Extracellular, granule-like deposits were scattered outside the perikarya in the ALS mutant lines. Boxed areas in the upper panels are enlarged in the lower panels. (**b**) Changes in the distribution of OPN-immunopositive signals during the disease course in SOD1^G93A^ mice. OPN-positive extracellular granules became detectable around the time of disease onset (P100) and increased in number as the disease progressed (P120–P170). (**c**) Western blot analysis of spinal cord lysates from non-transgenic control (WT) and SOD1^G93A^ mice (G93A) prepared at the indicated times. Representative blot out of three experiments. Full-length gels are shown in Supplementary Fig. 10a. (**d**) Double-immunofluorescent labeling of lumbar spinal cord sections from WT (P200) and SOD1^G93A^ (P140) mice shows the appearance of OPN-positive extracellular granules in the vicinity of ChAT-positive motor neurons in SOD1^G93A^ mice. Scale bar, 50 μm (**a** upper panels, **b**, **d**); 20 μm (**a** lower panels). See also Supplemental Fig. 3.

Spinal cord sections from three ALS mouse models (SOD1^G93A^, SOD1^G85R^, LoxSOD1^G37R^), human wild-type SOD1-expressing transgenics (WT-hSOD1) or non-transgenic controls (WT) were immunohistochemically analyzed for OPN. At the end stage of their respective diseases (P170 in SOD1^G93A^, P361 in SOD1^G85R^, P399 in LoxSOD1^G37R^), granular OPN-positive signals about 1–5 μm in diameter were scattered extracellularly (Fig. 3a). No similar granular OPN signals were detected in sections from WT on P200 (Fig. 3a) or WT-hSOD1 on P180 (Fig. 3a). Consistent with Fig. 1 and our earlier report^24^, OPN signals in WT mice were confined to the perikarya of a subset of α-MNs. The observed numbers of OPN-positive cells were smaller in all three ALS models than in WT mice, and the surviving OPN-positive cells in the mutant mice showed morphological signs of degeneration.

Figure 3b shows that before the onset of clinical disease (P60), the OPN signal was confined to MNs, and OPN-positive granules were rarely seen outside the perikarya, which was also the case for WT mice and WT-hSOD1 mice (Fig. 3a; ref. 24). Around the time of disease onset (P100), extracellular OPN-positive granules began to appear. Then during the period extending from P120 to P140, the numbers of OPN-positive granules increased until they were easily discernible. By the end stage of the disease (P170), only a few large OPN-positive perikarya were evident, but the density of the extracellular OPN deposits had increased greatly.

Western blot analysis (Fig. 3c) also showed that OPN levels within the spinal cord tissues did not change during the early stages of the disease (P100, P120), but had increased by a later stage (P150). The higher molecular weight bands (55–70 kDa) seen on P150 likely correspond to modified (e.g., phosphorylated and/or glycosylated) OPN species^36^. Fluorescent double labeling with anti-OPN and anti-ChAT antibodies showed that OPN signals from ChAT-positive motor neurons were diminished in late-stage disease and that there was a concomitant appearance of extracellular OPN granules (Fig. 3d).

To determine whether activated glial cells also began to express OPN during disease progression in ALS mouse models, we compared the distributions of OPN and glial markers (Iba1 for microglia/macrophages, GFAP for astrocytes) in spinal cord sections from SOD1^G93A^ or SOD1^G85R^ mice with late-stage disease (Supplementary Fig. 3a). In both models, a substantial portion of the OPN-positive granules were detected within or attached to Iba1-positive microglia/macrophages (>60%; 532/865 OPN granules counted for SOD1^G93A^). We detected colocalization of OPN and GFAP much less frequently (<2%; 14/824 OPN granules counted for SOD1^G93A^) (Supplementary Fig. 3a), and speculated that OPN is expressed in, or OPN granules are phagocytosed by, a subset of activated microglia/macrophages. To distinguish between these two possibilities, expression of OPN mRNA was analyzed using *in-situ* hybridization (ISH) along with Iba1 IHC (Supplementary Fig. 3b). In WT mice (200 days), OPN ISH signals were detected exclusively in MNs; Iba1-positive ramified microglia were devoid of OPN ISH signals. In SOD1^G93A^ mice (P170), we detected OPN mRNA in both MNs and Iba1-positive microglia (Supplementary Fig. 3b). Only a small fraction of the Iba1-positive cells was found to express OPN mRNA (7.8%; 60/767 cells counted), however, suggesting most of the OPN granules seen within Iba1-positive cells had been phagocytosed. In addition, double labeling of OPN and LAMP-1, a lysosome marker, revealed that LAMP-1-positive membrane structures were frequently associated with the phagocytosed OPN granules (Supplementary Fig. 3c), suggesting the granules were a form of phagolysosome.

**Figure 4:**
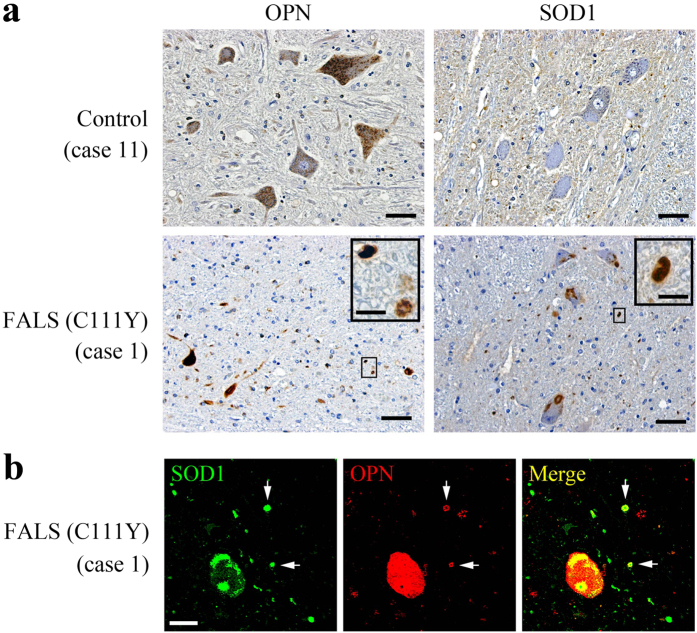
OPN is expressed in human motor neurons and detectable as granular deposits in a SOD1-linked FALS case. (**a**) OPN or SOD1 immunostaining in spinal cord ventral horn (L3 to L5) sections from a control or SOD1-linked FALS patient. OPN-positive signals were detected in the perikarya of motor neurons (control and FALS case) along with extracellular deposits in the FALS case. Both intracellular and extracellular SOD1 aggregates were detected in the FALS case. The boxed areas are enlarged in the inset panels. (**b**) Confocal analysis of OPN and SOD1 in the ventral spinal cord of the FALS case. The arrows indicate extracellular deposits positive for OPN/SOD1. Scale bar, 50 μm (**a**), 10 μm (inset in **a**), 20 μm (**b**).

The clinical phenotypes of FALS and SALS are essentially indistinguishable. To determine whether OPN is expressed in human MNs, and whether OPN-positive granules are present in ALS patients, we performed IHC studies using autopsied spinal cords from SOD1-linked FALS and SALS cases. The clinical characteristics of the patients used in this study are summarized in Supplementary Table 1. Detailed clinical and pathological analyses of a FALS family carrying a SOD1 C111Y mutation were reported elsewhere^37^,^38^. OPN signals were detected in ventral horn MNs in a control and SOD1-linked FALS case (Fig. 4a). Perikaryal OPN staining was also detected in the other two controls and eight SALS cases (not shown). Along with the intracellular staining, extracellular OPN-positive granular structures, around 5 μm in diameter, were detected in the FALS case (Fig. 4a). Typical SOD1-positive cytoplasmic and extracellular aggregates were seen in the FALS case, but not in the control cases (Fig. 4a). Fluorescent double labeling of the FALS sections revealed that extracellular OPN and SOD1 signals were often colocalized (Fig. 4b), whereas no extracellular OPN- or SOD1-positive granules were observed in any of the control or SALS cases analyzed so far (not shown).

### Increased proportion of OPN-high/MMP-9-high MNs in aged mice

Sarcopenia, the age-related reduction in muscle mass and strength, is caused by age-dependent loss of MNs and subsequent motor unit remodeling characterized by initial denervation of fast motor units followed by axonal sprouting and re-innervation by slower motor units^11^,^39^. To determine changes in OPN/MMP-9 expression can account for the MN degeneration seen with aging, we analyzed the OPN/MMP-9 expression profiles in MNs from 950-day-old male WT C57BL/6 mice, which have a median lifespan of 800 days. We found that the OPN-high/MMP-9-high MN fraction was much larger in the spinal cords of aged mice than young adults (P60) or adults (P200) (Fig. 1b, Supplementary Fig. 4a,b). Other than the γ-MNs (OPN-low/MMP-9-low; 40%), the largest MN fraction was the OPN-high/MMP-9-high group (24%), as the FF MN fraction (OPN-low/MMP-9-high) was halved (Supplementary Fig. 4b) as compared the corresponding MNs on P200 (Fig. 1b). However, we detected minimal expression of the ER stress markers ATF3 and P-eIF2α in these OPN-high/MMP-9-high and OPN-low/MMP-9-high MNs (data not shown), suggesting that aging leads to motor unit remodeling but not necessarily MN degeneration in the absence of mutant SOD1.

### Genetic ablation of OPN delays disease onset but accelerates disease progression in SOD1^G93A^ mice

**Figure 5:**
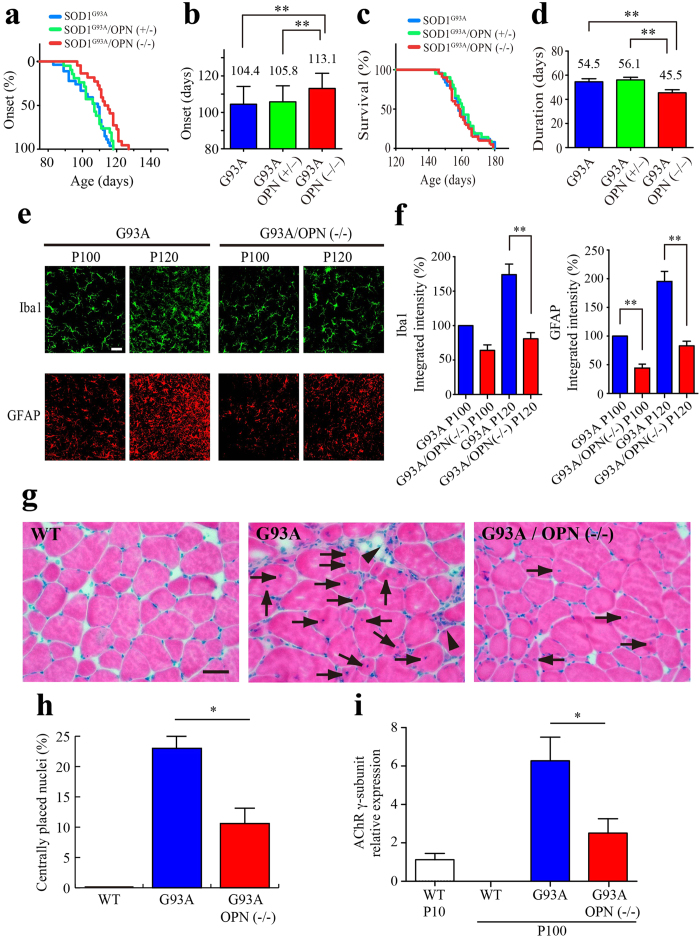
Genetic ablation of OPN delays disease onset but accelerates disease progression in SOD1^G93A^ mice. (**a**, **c**) Ages at disease onset (**a**) and disease end stage (**c**) among SOD^G93A^/OPN^−/−^ (red, n = 22), SOD1^G93A^/OPN^+/−^ (green, n = 21) and SOD1^G93A^ littermates (blue, n = 27). (**b**) Mean onset time (days ± SEM) in SOD^G93A^/OPN^−/−^ mice was significantly delayed (**p < 0.01, two-tailed Student’s test) as compared to SOD1^G93A^/OPN^+/−^ or SOD1^G93A^ mice (also statistically significant (p < 0.01) by Log-rank test). (**d**) Disease duration (from onset to end stage) was significantly shorter in SOD^G93A^/OPN^−/−^ (**p < 0.01, two-tailed Student’s test) than SOD1^G93A^/OPN^+/−^ or SOD1^G93A^ mice. (**e**) Images of Iba1 staining (green, microglia) or GFAP staining (red, astrocytes) in the lumbar spinal cord of SOD1^G93A^ and SOD^G93A^/OPN^−/−^ mice. (**f**) Relative expression of Iba1 or GFAP (5 sections per mouse, n = 3 for each genotype and age, **p < 0.01, two-tailed Student’s test) determined from integrated fluorescent intensity measurements. (**g**) Hematoxylin and eosin staining of gastrocnemius muscle (P100) from control (WT), SOD1^G93A^ and SOD^G93A^/OPN^−/−^ mice. The arrowheads indicate muscle atrophy, and the arrows denote centrally placed nuclei. (**h**) Frequency of centrally placed nuclei (8–10 sections per mouse, n = 3 for each genotype, *p < 0.05, two-tailed Student’s test). (**i**) Relative levels of AChR γ-subunit mRNA in gastrocnemius muscle from mice with the indicated genotypes and ages (n = 3). Data were obtained using qRT-PCR and are plotted relative to the expression in neonatal WT muscle (P10). *p < 0.05, two-tailed Student’s test. Scale bar, 20 μm (**e**), 50 μm (**g**).

Selective expression of OPN in ALS-resistant FR/S MNs and ECM accumulation in both the FALS case and three lines of SOD1-ALS model mice prompted us to test whether OPN is neuroprotective against motor neuron disease. We crossbred SOD1^G93A^ with OPN^−/−^ mice, which are reported to be grossly healthy with normal life spans^40^. The SOD1^G93A^/OPN^−/−^ and SOD1^G93A^/OPN^+/−^ mice were then compared to their SOD1^G93A^/OPN^+/+^ littermates with respect to time of disease onset, determined as the time of peak weight, and disease duration, defined as the period from peak weight until death (see Experimental Design in EXPERIMENTAL PROCEDURES). Surprisingly, SOD1^G93A^/OPN^−/−^ mice showed delayed disease onset as compared to SOD1^G93A^ or SOD1^G93A^/OPN^+/–^ mice (Fig. 5a,b), but significantly accelerated disease progression (Fig. 5d). As a result, we observed no difference in the overall life span of the mice (Fig. 5c).

The observed delay of disease onset was further confirmed based on various indexes, including grip-strength, glial hypertrophy, glial marker expression and muscle degeneration (Fig. 5e–i, Supplementary Fig. 5). We measured the forelimb grip strength of each mouse from P60 onward. Because in our hands the variability of the measured values was large, we increased the number of mice and analyzed the results using nonlinear regression (Supplementary Fig. 5). There was a gradual decline in grip strength in both SOD1^G93A^ and SOD1^G93A^/OPN^+/–^ mice. In SOD1^G93A^/OPN^−/−^ mice, however, we observed a plateau phase (P100–P120) during which the decline in grip strength was temporally paused, followed by a rapid decline (P120–P150).

Astrogliosis and microgliosis are two key features of ALS models and patients^15^,^16^. IHC analysis revealed both to be diminished in the ventral spinal regions of SOD1^G93A^/OPN^−/−^ mice, as compared to SOD1^G93A^ mice on P100 and P120 (Fig. 5e,f). In addition, angular muscle fibers and centrally placed nuclei, two signs of muscle degeneration, were frequently observed in the medial gastrocnemius muscles of SOD1^G93A^ mice on P100 (Fig. 5g), but were less evident in SOD1^G93A^/OPN^−/−^ mice (Fig. 5h). Moreover, real-time PCR analysis of AChR γ-subunit mRNA, which encodes an embryonic subunit replaced by the adult-type ε-subunit during postnatal muscle development^41^,^42^, revealed that muscle degeneration (reappearance of AChR γ-subunit mRNA) was milder in SOD1^G93A^/OPN^−/−^ than SOD1^G93A^ on P100 (Fig. 5i).

In order to functionally analyze whether NMJs of the MMP-9-expressed remodeled FR/S motor units degenerate slower in SOD1^G93A^/OPN^−/−^ mice compared with SOD1^G93A^ mice, gastrocnemius muscle was injected with fast blue at P97 and the retrogradely labeled MNs were counted at P100. Although statistical significance was not reached, there was a trend of increased number of fast blue-labeled MNs in SOD1^G93A^/OPN^−/−^ mice when compared with SOD1^G93A^ mice (Supplementary Fig. 6a). When the retrograde-labeled MNs were subdivided into MMP-9-high (FF or remodeled FR/S) or MMP-9-low (FR/S) subtypes, an increased number of fast blue (+)/MMP-9-high MNs was detected in SOD1^G93A^/OPN^−/−^ mice compared with SOD1^G93A^ mice (Supplementary Fig. 6b) with a statistical significance; however, there was still no statistical difference in the fast blue (+)/MMP-9-low MN population (Supplementary Fig. 6c). The results suggest that the MMP-9-expressed remodeled FR/S MNs survive longer in the absence of OPN.

### OPN stimulates MMP-9 expression through αvβ3 integrin

OPN reportedly enhances MMP-9 transcription and activation by binding αvβ3 integrin^27^,^28^,^29^,^30^,^31^. The accumulation of OPN in the ECM during the ALS progression (Figs 3 and 4) prompted us to study possible autocrine or paracrine actions on MNs mediated via OPN’s receptors, αvβ3 integrin and/or CD44^43^,^44^. Consistent with OPN-mediated enhancement of MMP-9 expression, in SOD1^G93A^ mice on P60, >80% of OPN-high/MMP-9-high MNs also showed immunoreactivity for β3 integrin (Supplementary Fig. 7a,b).

Similar induction of MMP-9 along with other neuronal differentiation markers (e.g., synapsin I and voltage-gated Na^+^ channels) was observed in ChAT-transfected N18TG2 neuroblastoma cells^45^. In the present study, Western blotting of culture medium conditioned by Neuro2a cells detected little MMP-9 expression, whereas ChAT-transfected Neuro2a (Neuro2a-ChAT) cells expressed detectable levels of MMP-9 (Supplementary Fig. 7c) and displayed a more differentiated morphology with enhanced neurite elongation (Supplementary Fig. 7d). Zymographic analysis of culture medium from Neuro2a-ChAT cells showed that OPN treatment induced dose- and time-dependent up-regulation of MMP-9 (Supplementary Fig. 7e,f), and that pretreating the cells with an anti-αv integrin neutralizing antibody (IM7) inhibited the OPN-induced increase in MMP-9 (Supplementary Fig. 7g). This confirms that an αvβ3 integrin-mediated autocrine loop mediates OPN-induced up-regulation of MMP-9 in Neuro2a-ChAT cells.

### CD44-mediated effects of OPN on astrocytes

**Figure 6:**
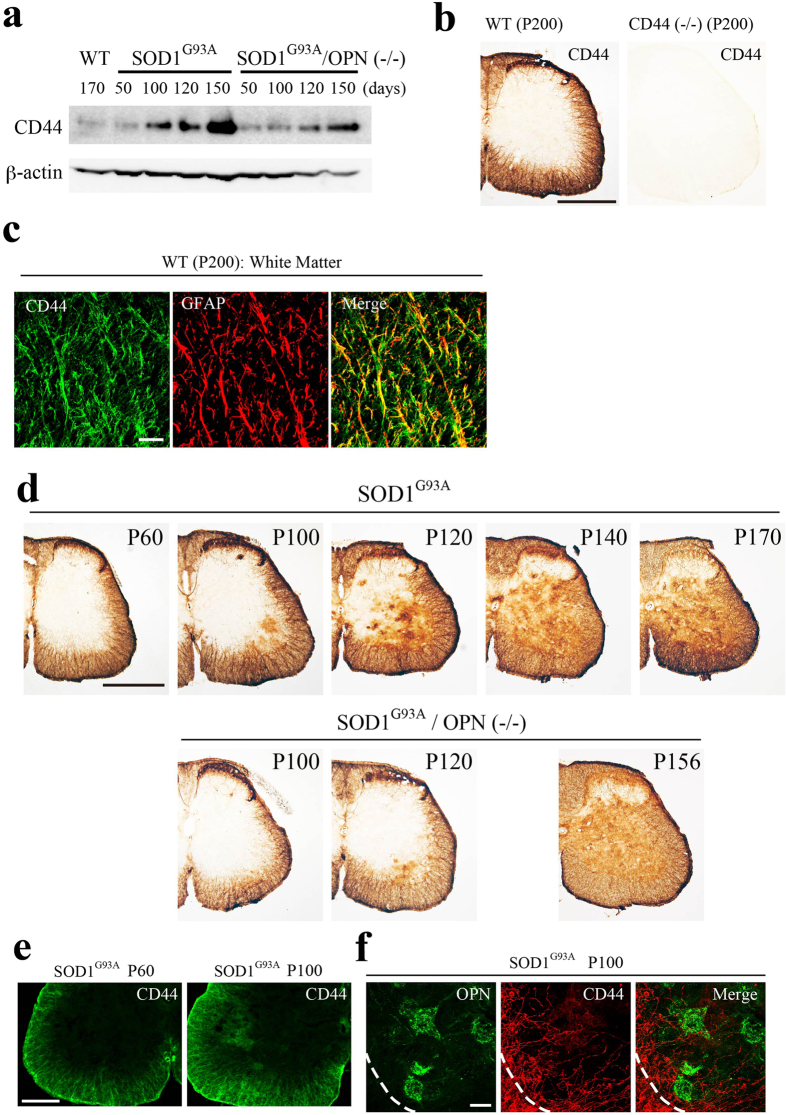
Changes in astrocytic CD44 expression during ALS disease progression in SOD1^G93A^ mice. (**a**) Western blot analysis of spinal cord lysates from non-transgenic control (WT), SOD1^G93A^ and SOD^G93A^/OPN^−/−^ mice prepared at the indicated disease stages (days). Increased CD44 expression was observed during disease progression in SOD1^G93A^ or SOD^G93A^/OPN^−/−^ mice. The full-length gel was shown in Supplementary Fig. 10b. (**b**) Immunostaining for CD44 (clone KM201) in WT mice revealed expression to be restricted to the white matter of the lumbar spinal cord. There was no staining in CD44^−/−^ mice. (**c**) Confocal images of CD44 (green) and GFAP (red) in the ventral white matter of the lumbar spinal cords in WT mice. CD44 staining significantly overlapped fibrous GFAP-positive astrocytes in the white matter. (**d**) Comparison of CD44 expression between SOD1^G93A^ and SOD^G93A^/OPN^−/−^ mice during the disease course. In SOD1^G93A^ mice, from P100 onward, patchy CD44 staining appeared in the ventral gray matter around the MN pool region, and then gradually spread dorsally. The appearance and spreading of CD44 in the white matter was delayed in SOD^G93A^/OPN^−/−^ mice as compared with SOD1^G93A^ mice. (**e**, **f**) Patchy spreading of CD44 staining from the white to the gray matter (**e**), and to the MN pool, where OPN-positive MNs are located (**f**) in SOD1^G93A^ mice. Dotted lines represent the white/gray matter boundary. Scale bar, 500 μm (**b**, **d**); 20 μm (**c**); 200 μm (**e**); 50 μm (**f**). See also Supplemental Fig. 8.

Because MN-derived OPN could potentially impact glial activation, we tested whether either of OPN’s receptors, αvβ3 integrin or CD44, are involved in OPN-induced astrocyte activation in SOD1^G93A^ or SOD1^G93A^/OPN^−/−^ mice. In both models, expression of CD44 mRNA (data not shown) and protein (Fig. 6a) increased during ALS disease progression. In WT mice, CD44 expression was restricted in fibrous astrocytes within the spinal white matter (Fig. 6b,c). Oligodendrocytes and their precursors identified using their respective markers (S100β and Olig2) were negative for OPN (data not shown). In the gray matter of SOD1^G93A^ mice, CD44-positive signals first appeared in the area of MN pools in the ventral cord around the time of disease onset (P100), and then extended dorsally to areas outside the MN pools (Fig. 6d). The spread of CD44 in the gray matter of SOD1^G93A^/OPN^−/−^ cords lagged behind that in SOD1^G93A^ cords at the same disease stage (Fig. 6d). The time course of the disease-associated changes in CD44 expression was then confirmed using an antibody recognizing a different CD44 epitope (Supplementary Fig. 8a). At the white/gray matter boundary on P100, CD44-positive fibrous astrocytes were found to migrate from the white matter into the MN pools (Fig. 6e,f). On the other hand, real-time PCR revealed no change in the expression of αv integrin mRNA during the course of ALS (data not shown). IHC staining using an anti-β3 integrin antibody revealed its expression to be MN-restricted in SOD1^G93A^ mice, even at an advanced stage of the disease (P140) (Supplementary Fig. 8b).

**Figure 7:**
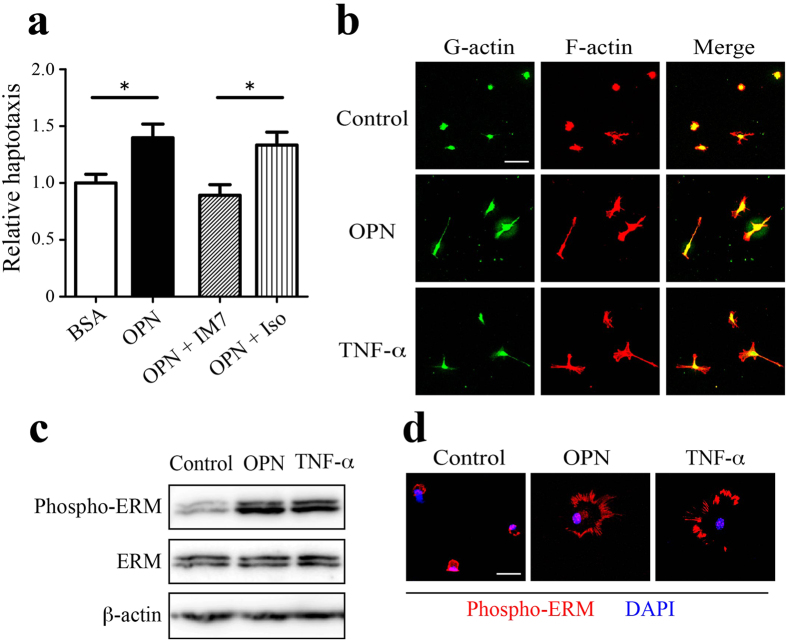
OPN induces CD44-mediated migration and morphological changes in cultured astrocytes. (**a**) Astrocyte haptotaxis toward OPN was mediated by CD44. The lower surface of migration chamber filters was coated with BSA (control) or OPN. Cultured astrocytes were added to the upper side of the migration chamber filters, and cells that migrated to the lower side were counted. Astrocyte haptotaxis toward OPN was inhibited by an anti-CD44 neutralizing antibody (IM7) but not by an isotype control antibody (Iso). Each bar represents the mean ± SEM relative to the mean of BSA-coated filters (n = 13–14). *p < 0.05, two-tailed Student’s test. (**b**) OPN induces morphological changes in cultured astrocytes. Primary astrocytes were cultured for 24 h in the absence (Control) or presence of OPN (1 μg/mL) or TNF-α (50 ng/mL), and labeled with DNase I (green) or phalloidin (red) to visualize G-actin and F-actin, respectively. Cell hypertrophy with extension of filopodia was evident in the OPN- or TNF-α-treated astrocytes. (**c**, **d**) OPN induces ERM activation. Primary astrocytes were treated with OPN or TNF-α as described above. Western blot analysis using an anti-phospho-ERM antibody revealed enhanced phosphorylation of ERM by OPN or TNF-α. Full-length gels are shown in Supplementary Fig. 10c. Representative blot of out of three experiments. Fluorescent phospho-ERM signals (red) were detected in filopodia-like leading edges of astrocytes treated with OPN or TNF-α. Scale bar, 50 μm (**b**); 20 μm (**d**).

To determine whether ECM-associated OPN directly facilitates astrocyte migration, haptotaxis assays were conducted using modified Boyden chambers in which the undersides of the chamber membranes were coated with target proteins. Immobilized OPN promoted a 1.5-fold increase in the migration of cultured astrocytes as compared to BSA-coated membranes. This OPN-induced haptotaxis was completely inhibited by an anti-CD44 neutralizing antibody (IM7), but not by an isotype control antibody (Fig. 7a). In addition, primary astrocytes were cultured in the presence or absence of OPN (or TNF-α, as a positive control), and their cell morphology was analyzed using isoform-specific probes for G- or F-actin. OPN, like TNF-α, induced cell hypertrophy with formation of F-actin-rich pseudopod-like protrusions (Fig. 7b). Western blotting reveled that OPN or TNF-α increased the phosphorylation of ezrin-radixin-moesin (ERM) proteins (Fig. 7c), which are known to connect CD44 to the actin cytoskeleton and promote cell motility^46^. Immunocytochemical (ICC) analysis of OPN- and TNF-α-treated cells demonstrated the presence of phospho-ERM within the pseudopod-like protrusions at the cell periphery (Fig. 7d). These OPN-induced changes in astrocyte morphology and ERM phosphorylation were completely blocked by an anti-CD44 neutralizing antibody (IM7; data not shown).

### Opsonin effect of OPN on microglial function

**Figure 8:**
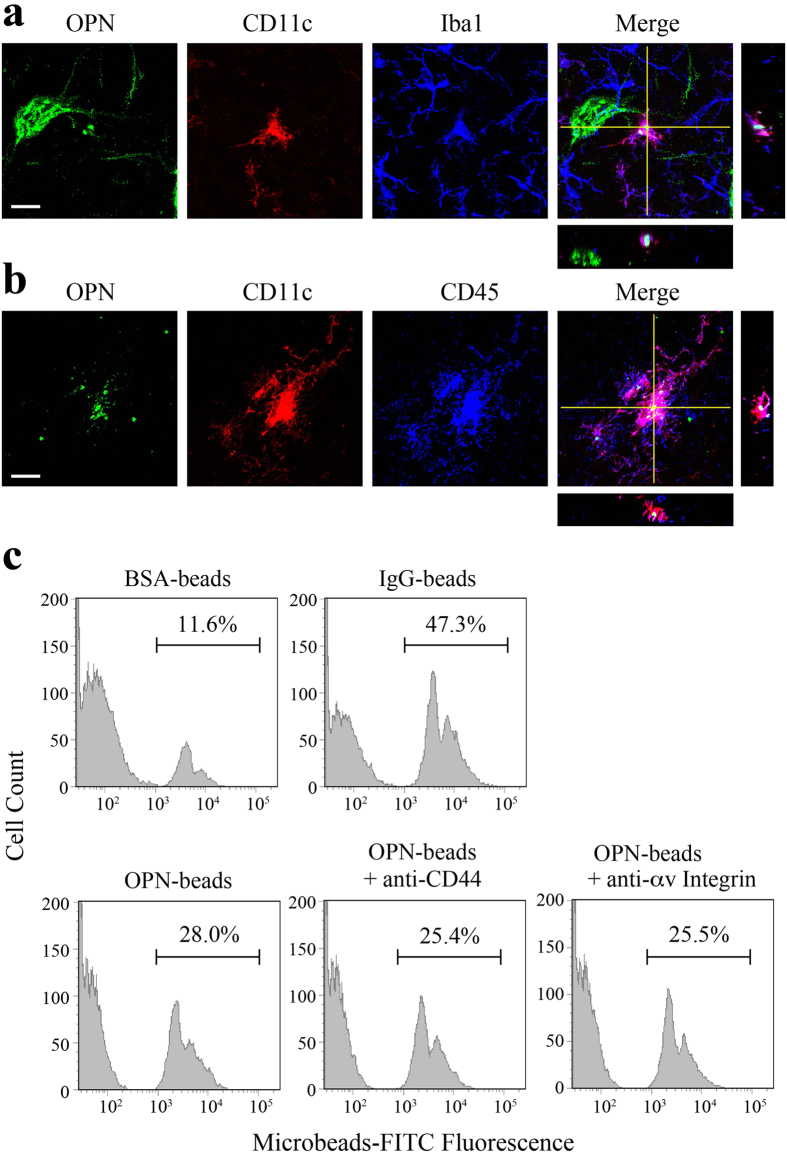
OPN acts as an opsonin facilitating phagocytosis by microglia/macrophages. (**a**) Triple immunostaining of OPN (green), CD11c (red) and Iba1 (blue) shows that OPN-granules are phagocytosed by microglia/macrophages, which are positive for CD11c in the ventral horn of SOD1^G93A^ mice on P100. (**b**) The OPN-phagocytosing and CD11c-positive cells are also positive for CD45 in the ventral horn of SOD1^G93A^ mice on P100. (**c**) Fluorescent microbeads coated with BSA, IgG or OPN were presented to cultured microglia, after which internalization of the beads by the microglia was analyzed and quantified using flow cytometry. BSA-coated and IgG-coated beads were used as negative and positive controls, respectively. Note that phagocytosis of OPN-coated beads was not influenced by prior treatment with anti-CD44 or anti-αv integrin neutralizing antibodies. Scale bar, 20 μm (**a**). See also Supplemental Fig. 9.

OPN reportedly functions as an opsonin facilitating phagocytosis by monocytes and macrophages^47^,^48^. We observed that OPN-positive granular deposits were actively phagocytosed by Iba1-positive cells in the spinal cords of ALS mice at late disease stages (Fig. 3). We therefore assessed OPN phagocytosis around the time of clinical disease onset (P100). In the vicinity of MNs, we often observed Iba1-positive cells containing internalized OPN-positive granules. These cells were also positive for CD11c (Fig. 8a), a phagocytosis marker and αx integrin, a recently identified OPN receptor^48^, as well as CD45 (Fig. 8b), which suggests these cells were not dendritic cells but were microglia or macrophages. Further IHC analysis using a panel of antibodies revealed that the OPN-phagocytosing microglia/macrophages did not conform to the typical M1 or M2 phenotype. These atypical cells were positive for CD68 (Supplementary Fig. 9a), CD86 (Supplementary Fig. 9b) and MHC class II (Supplementary Fig. 9c), but were negative for iNOS, NOX2 and CD206 (data not known).

To evaluate the opsonization effect of OPN *in vitro*, fluorescent beads (3 μm in diameter) were covalently coupled with OPN, BSA (negative control) or IgG (positive control) and presented to cultured primary microglia, after which internalization of the beads was analyzed using flow cytometry (Fig. 8c). The OPN-coated beads (OPN-beads) were more efficiently phagocytosed by cultured microglia than the negative control (BSA-beads). The facilitative effect of OPN on microglial phagocytic activity was not suppressed by an anti-CD44 or anti-αv integrin neutralizing antibody, suggesting OPN-mediated phagocytosis is independent of both those proteins.

## Discussion

Distinguishing early disease-causing dysfunction from similar adaptive responses constitutes a major challenge in ALS research. In rodent models of ALS, early disease-associated changes, such as axonal dysfunction, mitochondrial abnormality and ER stress, occur long before the onset of symptomatic disease^8^. Moreover, preclinical neuromuscular abnormalities reflecting loss of FF MNs are compensated for through adaptive axonal sprouting and re-innervation by FR or S MNs^9^,^13^,^49^. In the present study, we found that FR/S MNs in SOD1^G93A^ mice gain FF MN-like characteristics through expression of MMP-9. There is also evidence that changing muscle phenotypes through expression of myogenic factors^50^ or microRNA^51^ has an impact on disease progression in SOD1^G93A^ mice. Consistent with that idea, it was recently reported that muscle contributes to the progression of MN degeneration in a BAC mouse model of X-linked spinal and bulbar muscular atrophy^52^.

Sarcopenia, the age-related loss of skeletal muscle mass and strength, is caused by myofiber denervation^53^,^54^. We found that, in the spinal cords of old mice (P950), the OPN-high/MMP-9-high MN fraction was increased with a concomitant decrease in the OPN-low/MMP-9-high fraction (Supplementary Fig. 4). Although this implies there could be common defects in ALS and sarcopenia, in old mice the OPN-high/MMP-9-high MNs were negative for ER-stress markers, which suggests simple co-expression of OPN and MMP-9 does not necessarily induce neurodegeneration. Nonetheless, through comparison of NMJs in normally aging and SOD1^G93A^ mice, Valdez *et al.*^55^ observed that there are striking similarities between the two conditions, and that normal aging affects MN subtypes in the same vulnerability sequence (FF to FR/S MN) as typically seen in ALS. Thus the appearance of OPN-high/MMP-9-high MNs in aging mice may reflect the active remodeling of NMJs as is observed in SOD1^G93A^ mice.

OPN is expressed in a variety of cell types, including neurons, where it promotes neuronal survival and regeneration^56^,^57^,^58^,^59^. We suspected that OPN expressed in FR/S MNs might render them resistant to mutant SOD1-induced toxicity. To test that idea, we crossbred SOD1^G93A^ with OPN^−/−^ mice, and analyzed their offspring. Surprisingly, disease onset was delayed, but disease progression is accelerated, reflecting two modes of OPN action, which we suggest are respectively cell-autonomous and non-cell-autonomous. The cell-autonomous action of OPN is mediated by αvβ3 integrin and induces up-regulation of MMP-9, while the non-cell-autonomous action activates astrocytes and microglia, possibly making their phenotypes neuroprotective.

Analyzing the non-cell-autonomous action of OPN may consist an important necessary step for developing a novel ALS therapy. We observed migration of CD44-positive astrocytes and OPN-phagocytosing microglia/macrophages within MN pools over the ALS disease course in SOD1^G93A^ mice. We speculate that OPN triggers neuroinflammation in the affected MN areas, which may explain, at least in part, the ALS-specific spread of pathological transformation. In addition, CD44 expression was increased early in the affected MN areas and later in the entire spinal cord, and the timing of the increment in CD44 expression was delayed by genetic ablation of OPN (Fig. 6, Supplementary Fig. 8). *In vitro* haptotaxis assays showed that an anti-CD44 neutralizing antibody inhibited OPN-induced migration of primary cultured astrocytes (Fig. 7a). We therefore conclude that OPN-induced astrocyte migration and invasion into affected MN areas is mediated by CD44.

We also found that OPN-phagocytosing microglia/macrophages detected around the time of disease onset do not conform to the typical M1 or M2 phenotype, and that OPN-induced activation of microglia/macrophages may involve a mechanism exhibiting both site- and time-dependency. Although the receptors responsible for OPN-induced phagocytosis by microglia/macrophages are not yet known, our findings suggest that neither αvβ3 integrin nor CD44 is involved. We suggest αxβ2 integrin (CD11c), a novel OPN receptor identified by Schack *et al.*^48^, is responsible, as the OPN-phagocytosing microglia/macrophages are clearly CD11c-positive (Fig. 8, Supplementary Fig. 9). Characterization of the cell-autonomous and non-cell-autonomous actions of OPN will require detailed analysis involving cell-type-specific genetic ablation of OPN in mutant SOD1 mice, however.

A major challenge in the treatment of ALS is to delay disease progression after clinical onset. Our results suggest the OPN-αvβ3 integrin-MMP-9 axis is a potentially useful target for ALS therapy. Because this axis is involved in the second wave of neurodegeneration in ALS, intervention may help to slow the progression of neuromuscular symptoms in cases where a valid early diagnosis is available. Targeting OPN itself may be less than optimal, since although blocking OPN action may slow FR/S MN degeneration, and so delay manifestation of clinical ALS symptoms, it may also inhibit OPN’s protective action via glial cells. Another possibility is to inhibit MMP-9. Broad spectrum or selective MMP inhibitors have long been targeted for drug development, but without success in clinical trials, possibly due to dose-limiting side effects^60^. Nonetheless, the positive effect of MMP-9 inhibitor I on SOD1^G93A^ mice^32^ suggests MMP-9 inhibition may be a promising approach to ALS treatment, once the risks of adverse side effects are overcome.

We propose that by slowing OPN-mediated degeneration of FR/S MNs, inhibition of αvβ3 integrin may also be an effective strategy for treating ALS. We observed expression of αvβ3 integrin in MNs vulnerable to ALS (Supplementary Fig. 7a,b), and its expression is restricted to MNs, with minimal expression in glial cells, even at advanced stages of the disease. By selectively inhibiting OPN action on MNs, inhibitors of αvβ3 integrin may delay the onset and progression of ALS without inhibiting potentially protective effects of OPN on glial cells. It is noteworthy that OPN effects on MNs are mediated via typical RGD-type ligand binding to αvβ3 integrin, but those on glial cells are mediated by non-RGD-type binding through CD44 or αx integrin^43^,^48^.

Wright *et al.*^59^ recently reported that OPN is up-regulated in Schwann cells (SCs) surrounding injured motor axons and help guide regenerating motor axons toward target muscles both *in vitro* and *in vivo*. They suggested that motor neuron regeneration is diminished in OPN^−/−^ mice as compared to OPN^+/+^ mice. Although they do not address ALS in that study, SC-expressed OPN may serve as another disease-modifying factor affecting peripheral axonal remodeling during the course of ALS, perhaps by facilitating compensatory remodeling of denatured motor axons. More recently, Capote *et al.*^61^ reported that OPN is expressed in dystrophic muscles in mdx mice, a model of Duchenne muscular dystrophy, and OPN ablation in the mice (OPN^−/−^mdx) promotes muscle repair by altering infiltrating macrophages toward a more pro-regenerative phenotype. It will be interesting to analyze OPN expression in muscle and infiltrating immune cells in the context of mutant SOD1-linked ALS models. Taken together, these findings and ours suggest that OPN is expressed in MN axons, SC endoneurial tubes and dystrophic muscles. Further investigation into the function of OPN, under normal and disease conditions, and its potential influence on the interaction amongst these cells is needed.

In summary, we found that OPN acts as an FR/S-type MN marker and a disease modifier that exerts selective cell-autonomous and non-cell-autonomous effects on MN vulnerability in SOD1^G93A^ mice. OPN is involved in the second wave of neurodegeneration in ALS, during which MMP-9 makes ALS-resistant FR/S MNs vulnerable to SOD1-mediated toxicity around the time of clinical disease onset. The OPN-αvβ3 integrin-MMP-9 axis may offer a target for ALS therapy aimed at inhibiting locally recruited disease-propagating processes.

## Experimental Procedures

### Mice

Transgenic mice carrying the human mutant SOD1^G93A^ gene (B6.Cg-Tg(SOD1*G93A)1Gur/J), mice with targeted disruption of the OPN gene (B6.129S6(Cg)-Spp1^tm1Blh^/J), and mice with targeted disruption of the CD44 gene (B6.129(Cg)-Cd44^tm1Hbg^/J) were purchased from Jackson Laboratory. Mice carrying the human SOD1^G85R^ gene (line 148) or the wild-type human SOD1 gene (B6.Cg-Tg(SOD1)76Dpr) were kind gifts from Dr. Don Cleveland (University of California, San Diego). LoxSOD1^G37R^ mice were described previously^62^. Mice were genotyped for human SOD1 as described^62^.

### Experimental Design

All animals were on C57BL/6 background. Heterozygous male SOD1^G93A^ mice were crossed with mice heterozygous for the OPN gene to generate SOD1^G93A^/OPN^−/−^ mice as F2 litters. The OPN alleles were genotyped as described previously^40^. Genotyping primers are shown in Supplementary Table 2. To analyze mouse phenotype and survival, SOD1^G93A^/OPN^−/−^ mice were always compared with their SOD1^G93A^/OPN^+/−^ or SOD1^G93A^/OPN^+/+^ littermates. Time of disease onset was retrospectively determined as the time when mice reached peak body weight. Disease end-stage was defined as the time at which the animal could not right itself within 30 s after being placed on its side. Disease duration was defined by the period between the onset and disease end-stage. All experiments were performed in accordance with Japanese national guidelines and regulations, and were reviewed and approved by the Animal Care and Use Committees of Keio University and Nagoya University, and care was taken to minimize suffering and limit the number of animals used. The number of animals used were shown in each of the experiments. Sexes were balanced in each experimental group when analyzing life-span and motor performance to avoid possible bias originating from sex-related intrinsic disease severity^63^. Aged male C57BL/6 mice (P944-P950) were obtained from the Tokyo Metropolitan Institute of Gerontology, where a specific pathogen-free aging colony is maintained.

### Human tissue samples

Spinal cord specimens from a patient with FALS and carrying an SOD1 mutation (C111Y) and six patients with sporadic SALS were obtained at autopsy. The diagnosis of ALS was made based on the El Escorial diagnostic criteria. Spinal cord tissues from three individuals with no history of neurological disease were used as normal control samples (NC). The study was performed in accordance with Japanese national guidelines and regulations, and was reviewed and approved by the human ethics committees of Keio University Faculty of Pharmacy, National Chushin-Matsumoto Hospital and Tokyo Metropolitan Geriatric Hospital and Institute of Gerontology. Informed consent was obtained from all individuals or their guardians before autopsies. For IHC staining, sections were processed as described previously^38^.

### Immunohistochemistry

Mice were deeply anesthetized with sodium pentobarbital and then perfused via the aortic cone with phosphate-buffered saline (PBS), followed by 4% paraformaldehyde (PFA) in 0.1 M phosphate buffer at pH 7.4 (PB). The lumbar region of each spinal cord (ca. 2 cm) was sampled and postfixed in the same fixative overnight at 4 °C, after which it was immersed in 20% sucrose in PB overnight at 4 °C. The tissue was then frozen in OCT compound (Sakura Finetek), and the L2 to L5 lumbar region of the spinal cord was serially sectioned at 40 μm using a cryostat. One in six free-floating sections were immunohistochemically processed as described previously^64^. For light microscopy, diaminobenzidine (DAB) was used as the chromogen. For epifluorescence microscopy and confocal scanning microscopy, sections were incubated with primary antibodies and then with Alexa Fluor-conjugated secondary antibodies (1:200 dilution; Invitrogen) and wet-mounted in Fluoromount-G (Southern Biotechnology). The primary antibodies and dilutions used were as follows: goat polyclonal anti-OPN (1:50,000 for peroxidase, 1:1,000 for fluorescence; AF808, R&D Systems), mouse monoclonal anti-OPN (1:5,000 for peroxidase, 1:500 for fluorescence; clone Akm2A1, sc-21742, Santa Cruz Biotechnology), goat polyclonal anti-MMP-9 (1:500 for fluorescence; M9570, Sigma), rabbit polyclonal anti-Iba1 (0.1 μg/ml for peroxidase, 0.2 μg/ml for fluorescence; 019-19741, Wako Pure Chemical Industries), rabbit polyclonal anti-GFAP (1:5,000 for fluorescence; Z0334, Dako), rabbit polyclonal anti-ChAT (1:2,000 for fluorescence, ref. 61), rat monoclonal anti-LAMP-1 (1:2,000 for fluorescence; clone 1D4B, 121601, BioLegend), rabbit monoclonal anti-β3 integrin (1:1,500 for fluorescence; EPR2417Y, Abcam), rabbit monoclonal anti-Phospho-eIF2α (Ser51) (1:250 for fluorescence; clone D9G8, Cell Signaling Technology), rabbit polyclonal anti-ATF-3 (1:500 for fluorescence; H-90, sc-22798, Santa Cruz Biotechnology), and FITC-labeled rat monoclonal anti-mouse CD45 (1:250; clone 30-F11, 11–0451, eBioscience). Double- or triple-labeled sections were examined using an Olympus BX51 microscope (Figs 3d and 6e) or an Olympus FV-1000 confocal microscope system (Tokyo, Japan). Confocal image stacks were constructed using a 60× objective lens (1.35 N.A., 2.0× optical zoom, 0.5 μm z-step) or 100× objective lens (1.40 N.A., 4.0× optical zoom, 0.43 μm z-step). Fluorescence intensity measurements were made by calculating the summed fluorescence intensity in a manually selected 100-μm^2^ region of interest (ROI) after subtracting the background. Human sections were analyzed using the following antibodies: goat polyclonal anti-human OPN (0.4 μg/ml for peroxidase, 2 μg/ml for fluorescence; K-20, sc-10591, Santa Cruz Biotechnology) and mouse monoclonal anti-human SOD1 (1 μg/ml for peroxidase, 5 μg/ml for fluorescence; clone 1G2, M062-3, MBL).

### Western blot and Zymography

Lumbar spinal cord segments were rapidly dissected from mice after cervical dislocation and homogenized in a lysis buffer (1% Triton X-100, 0.5% sodium deoxycholate, 0.1% SDS, 150 mM NaCl, 10 mM Tris-HCl, pH 7.5) supplemented with 1x protease inhibitor cocktail (Nakarai Tesque). After centrifugation for 15 min at 17,000 × g and 4 °C, aliquots of lysate containing 20 μg of protein were subjected to 10% SDS-polyacrylamide gel electrophoresis, and the separated proteins were transferred to polyvinylidene difluoride membranes (Immobilon-P; Millipore). The membranes were then blocked for 1 h at room temperature in Tris-buffered saline (TBS; 10 mM Tris-HCl, pH 7.5 and 150 mM NaCl) containing 0.05% Tween-20 and 5% nonfat skim milk. Once blocked, the membranes were incubated overnight at 4 °C with the following antibodies: rabbit polyclonal anti-mouse OPN (1:200 dilution; O-17, OK-516, IBL), goat polyclonal anti-MMP-9 (1:500 dilution; M9570, Sigma), rat monoclonal anti-CD44 (1:500; KM201, ab25340, Abcam) or mouse monoclonal anti-actin (1:1,000, clone C4, MAB1501R, Millipore). The membranes were then incubated with appropriate horseradish peroxidase-conjugated secondary antibodies (Bio-Rad). The bands were visualized using ECL Western Blotting Detection Reagent (GE Healthcare) and were captured using an ODYSSEY Fc Imaging System (LI-COR Biosciences).

Gelatin zymography was carried out on 10% SDS-PAGE gel copolymerized with 0.1% gelatin (Nacalai tesque). Gels were washed in renaturing buffer (2.5% Triton X-100) for 30 min at room temperature and then incubated in developing buffer (50 mM Tris-HCl, pH 8.5, 0.2 M NaCl, 5 mM CaCl_2_, 2% Triton X-100) for 24 h at room temperature. Staining was performed in 0.5% Coomassie blue G-250, followed by destaining in 40% methanol/10% acetic acid. Gelatinase standard was used as a MMP marker (AK38, Cosmo Bio).

### *In situ* hybridization combined with immunohistochemistry

Mice were deeply anesthetized with sodium pentobarbital and then perfused via the aortic cone with phosphate-buffered saline (PBS), followed by 4% PFA containing 0.2% saturated picric acid in PB. The lumbar region of the spinal cord was removed and postfixed in the same fixative for 2 h at 4 °C, after which it was dehydrated and embedded in paraffin. Sections were cut at 7 μm, mounted on MAS-coated slides (Matsunami Glass), and hybridized with digoxigenin (DIG)-labeled probes, as described previously^64^. To prepare the DIG-labeled OPN probe, a portion (189–985) of the mouse OPN sequence (NM_009263.3) was subcloned into pBluescript SK vector (Stratagene), and antisense and sense probes were transcribed using T7 and T3 RNA polymerase, respectively. The sense probe gave no hybridization signal. After color development using NBT/BCIP, sections were briefly washed in PBS and then processed for immunohistochemical detection of Iba1 using DAB as the chromogen, as described in Experimental Procedures.

### RNA isolation and qRT-PCR

Total RNA was extracted from spinal cords, muscle or primary glial cells using TRIzol (Life Technologies) according to the manufacturer’s instructions. Reverse transcription (5 μg of RNA) and quantitative PCR were performed with PrimeScript^TM^ RT Master Mix (Takara Bio) and THUNDERBIRD^TM^ SYBR qPCR Mix (Takara Bio) using a Thermal Cycler Dice Real Time System II (TP900; Takara Bio): initial denaturation at 95 °C for 30 s was followed by 40 cycles of 95 °C for 5 s, 60 °C for 1 min, and a final dissociation stage entailing 95 °C for 15 s, 60 °C for 15 s and 95 °C 15 s. Primer sequences are listed in Supplementary Table 2. Relative gene expression normalized to mouse GAPDH was determined using the ΔΔCt method. All reactions were conducted in duplicate.

### Motor neuron count

The L2 to L5 lumbar regions of spinal cords were collected and serially sectioned at 40 μm, after which one in six free-floating sections was stained for ChAT, a representative MN marker, along with other proteins. Only cells with a clearly visible nucleus and ChAT-positive cytoplasm located in the ventral horn were deemed to be MNs.

### Muscle analysis

Gastrocnemius muscles were freshly frozen in isopentane cooled in liquid nitrogen, after which transverse 20-μm cryostat sections were used for muscle immunohistochemistry and morphological analysis. After mounting on MAS-coated slides (Matsunami glass), the sections were fixed in cold acetone and processed for immunohistochemical staining as described above using mouse monoclonal antibodies to the type I, IIA and IIB myosin heavy chain (MyHC) isoforms (HB287, HB277 and HB283, respectively; ATCC). Hybridoma clones HB287 and HB277 were intraperitoneally injected into nude mice, and the ascites fluid was harvested and labeled with Alexa Fluor594 NHS Ester (Molecular Probes). For HB283, the IgM fraction was purified from culture supernatant using HiTrap IgM Purification HP (GE), and Alexa Fluor594-labeled donkey anti-mouse IgM, μ Chain Specific (Jackson ImmunoResearch Laboratories) was used as a secondary antibody for detection. For morphological analysis, cryosections were stained with hematoxylin and eosin.

### Grip strength

Forelimb grip strength was measured biweekly using a Mouse Grip Strength Meter 1027DSM (Columbus Instruments) starting on P60 (n = 14–21 for each genotype). The maximal value (given by Newton) from 5 trials on each day was adapted, and nonlinear regression analysis followed by curve fitting was performed by applying a four-dimensional polynomial model (GraphPad Prism5).

### Retrograde labeling of MNs

Local injection of fast blue into muscle and subsequent analysis of spinal cord sections were conducted as described previously^24^. Fluorescently labeled cells with a diameter greater than 20 μm and an identifiable nucleus were counted.

### Neuro2a-ChAT cells and OPN treatment

Mouse neuroblastoma Neuro2a cells were stably transfected with an expression vector containing mouse choline acetyltransferase (ChAT) cDNA (pEFmChAT), as described^65^. The G418-reresistant cell lines (Neuro2a-ChAT or Neuro2a-neo) were grown at 37 °C under a 5% CO_2_/95% air atmosphere in DMEM supplemented with 10% fetal bovine serum, 100 units/ml penicillin and 100 μg/ml streptomycin (Life Technologies). Neuro2a-ChAT cells seeded at 1.0 × 10^6 ^cells/well in 6-well plates were treated with various concentrations of recombinant mouse OPN (carrier-free, R&D Systems) for selected time periods. After incubation, the medium was collected, concentrated to 20×, and subjected to gelatin zymography as described above. For western blot analysis, cell lysate was prepared in RIPA buffer (1% Triton x-100, 0.5% sodium deoxycholate, 0.1% SDS, 150 mM NaCl, 10 mM Tris-HCl (pH 7.5) and 1x protease inhibitor cocktail. For morphological analysis, cultured cells on coverslips were fixed in 4% PFA in PBS and stained with mouse monoclonal anti-class III β-tubulin (1:1,000; clone TUJ1, Covance) and Alexa594-labeled secondary antibody.

### Primary glial culture

Primary microglia and astrocytes were obtained from mixed glial cultures. Cortexes from 1- to 2-day-old C57BL/6 mice were stripped of meninges and large blood vessels, minced with blades, dissociated with Trypsin and DNaseI, cleared using a 70-μm cell strainer (Falcon), and plated on poly-D-lysine-coated dishes in DMEM containing 10% FBS, 100 units/ml penicillin and 100 μg/ml streptomycin (Life Technologies). After 12 days, microglia were collected as floating cells after gentle shaking for 30 min. The remaining attached cells were deemed to be primary astrocytes. Immunocytochemical staining with Iba1 or GFAP antibody indicated >90% purity in each cell population. For morphological analysis, cells were seeded onto poly-D-lysine-coated, 9-mm ACLAR round coverslips (Allied Fibers & Plastics, Pottsville, PA, USA).

### Cell migration assay

Transwell migration chambers with 8-μm-pore polycarbonate membrane inserts (Costar) were used to assess cell migration. OPN, BSA or vitronectin (10 μg/mL in PBS, 24 h at room temperature) was applied to the bottom surface of each filter, which was then blocked with BSA (10 mg/ml in PBS, 1 h at room temperature) and then washed with DMEM. Human vitronectin was purchased from BD Biosciences. Primary cultured astrocytes (1 × 10^6 ^cells/well) were added to the upper chamber in migration assay buffer (DMEM containing 10 mg/mL BSA) and incubated for 24 h at 37 °C under a 5% CO_2_/95% air atmosphere. Cells on the upper filter surface were then removed with a cotton swab, and cells on the lower filter surface were stained with 0.2% crystal violet/2% ethanol followed by a brief rinse in distilled water. After drying the membrane, the number of cells that had migrated to the lower filter surface were determined in triplicate by counting cells in at least 10 randomly selected fields under a microscope. Anti-CD44 neutralizing antibody (IM7) and control rat IgG2b, κ isotype (1 μg/mL each) were purchased from BD Biosciences.

### Actin labeling and ERM phosphorylation in astrocytes

Primary cultured astrocytes grown on coverslips were incubated with recombinant mouse OPN (1 mg/mL) or recombinant human TNF-α (50 ng/mL: Pepro Tech) for 6 h at 37 °C under a 5% CO_2_/95% air atmosphere. To detect actin, the cells were washed with PBS, fixed in 4% PFA/0.1 M PB (pH 7.4) for 15 min at room temperature, and treated for 15 min with PBS containing 0.2% Triton X-100. G- and F-actin were then labeled using Alexa488-conjugated deoxyribonuclease I (1:500, Molecular Probes) and rohdamine-conjugated phalloidin (1:1,000, Invitrogen), respectively. For phospho-ERM labeling, the cells were washed with PBS containing 30 mM glycine (G-PBS), fixed with ice-cold 10% trichloroacetic acid for 15 min, and treated for 15 min with G-PBS containing 0.2% Triton X-100. After blocking the cells for 1 h in G-PBS containing 2% BSA and 3% normal goat serum, they were incubated for an additional 1 h with rabbit anti-phospho-ERM (1:400, Cell Signaling Technology). The immune-signals were then visualized using Alexa Fluor568-labeled goat anti-rabbit IgG (1:400, Molecular Probe), and nuclei were visualized using DAPI (1:1,000, Molecular Probe). For Western blot analyses, cell lysates were prepared in RIPA buffer containing 1x protease inhibitor cocktail as described above, and were probed with rabbit anti-ERM (1:1,000, Cell Signaling Technology) or anti-phospho-ERM.

### Covalent coupling of proteins to microsphere beads

Carboxylate-modified, FITC-marked, melamine resin microparticles (mean diameter, 3 μm; 1.4 × 10^8^ particles; Sigma-Aldrich) were mixed for 16 h at room temperature with OPN, BSA or IgG (each 20 μg) in a 0.2 mL volume of coupling buffer (25 mM phosphate buffer, pH 7.2 for OPN and BSA; 25 mM borate buffer, pH 8.5 for IgG). After washing five times with coupling buffer, the protein-coupled microspheres were resuspended in DMEM.

### Microglial phagocytosis and flow cytometry

Murine primary microglia prepared as described above were grown in DMEM containing 10% FBS, 100 units/ml penicillin and 100 μg/ml streptomycin. For phagocytosis assays, the cells were seeded at 8 × 10^5 ^cells/well into 6-well plates. After 24 h, the medium was replaced with DMEM containing 0.5% FBS, protein-coupled microspheres (10 beads/cell ratio) were added to each well, and the plates were incubated for 30 min at 37 °C in the presence or absence of an anti-CD44 (1 μg/mL; IM7) or anti-αv integrin neutralizing antibody (1 μg/mL; clone RMV-7, 550024, BD). The cells were then washed 3 times with cold PBS, collected in FACS buffer (PBS containing 2% FBS) and examined using flow cytometry in a LSR II Flow Cytometer running FACSDiva Software v8.0 (BD). A total of 10,000 events were collected from each sample.

### Statistical analysis

The data are presented as means ± standard error of the mean (SEM) unless otherwise indicated. The results were analyzed statistically using one-way analysis of variance (ANOVA) followed by Tukey-Kramer multiple comparison post hoc tests. Otherwise, unpaired Student’s t test was used to estimate differences between means. A log-rank test with Bonferroni corrections for multiple comparisons was used to calculate the statistical differences in the onset and survival of the different mouse cohorts. Data analyses were done using GraphPad Prism 5.0 (GraphPad Software). Differences were considered statistically significant when P values were less than 0.05.

## Additional Information

**How to cite this article**: Morisaki, Y. *et al.* Selective Expression of Osteopontin in ALS-resistant Motor Neurons is a Critical Determinant of Late Phase Neurodegeneration Mediated by Matrix Metalloproteinase-9. *Sci. Rep.* **6**, 27354; doi: 10.1038/srep27354 (2016).

## Supplementary Material

Supplementary Information
