# Supplementary material for: Selective Expression of Osteopontin in ALS-resistant Motor Neurons is a Critical Determinant of Late Phase Neurodegeneration Mediated by Matrix Metalloproteinase-9
Source: Sci Rep. 2016 Jun 6;6:27354. doi: 10.1038/srep27354 (PMC4893611; doi:10.1038/srep27354)
Supplement: Supplementary Information [file srep27354-s1.doc]

**Supplementary Information**

**(Supplementary Figures 1〜10, Supplementary Tables 1, 2)**

**Selective Expression of Osteopontin in ALS-resistant Motor Neurons is a Critical Determinant of Late Phase Neurodegeneration Mediated by Matrix Metalloproteinase-9**

Yuta Morisaki, Mamiko Niikura, Mizuho Watanabe, Kosuke Onishi, Shogo Tanabe, Yasuhiro Moriwaki, Takashi Okuda, Shinji Ohara, Shigeo Murayama, Masaki Takao, Sae Uchida, Koji Yamanaka, Hidemi Misawa

**
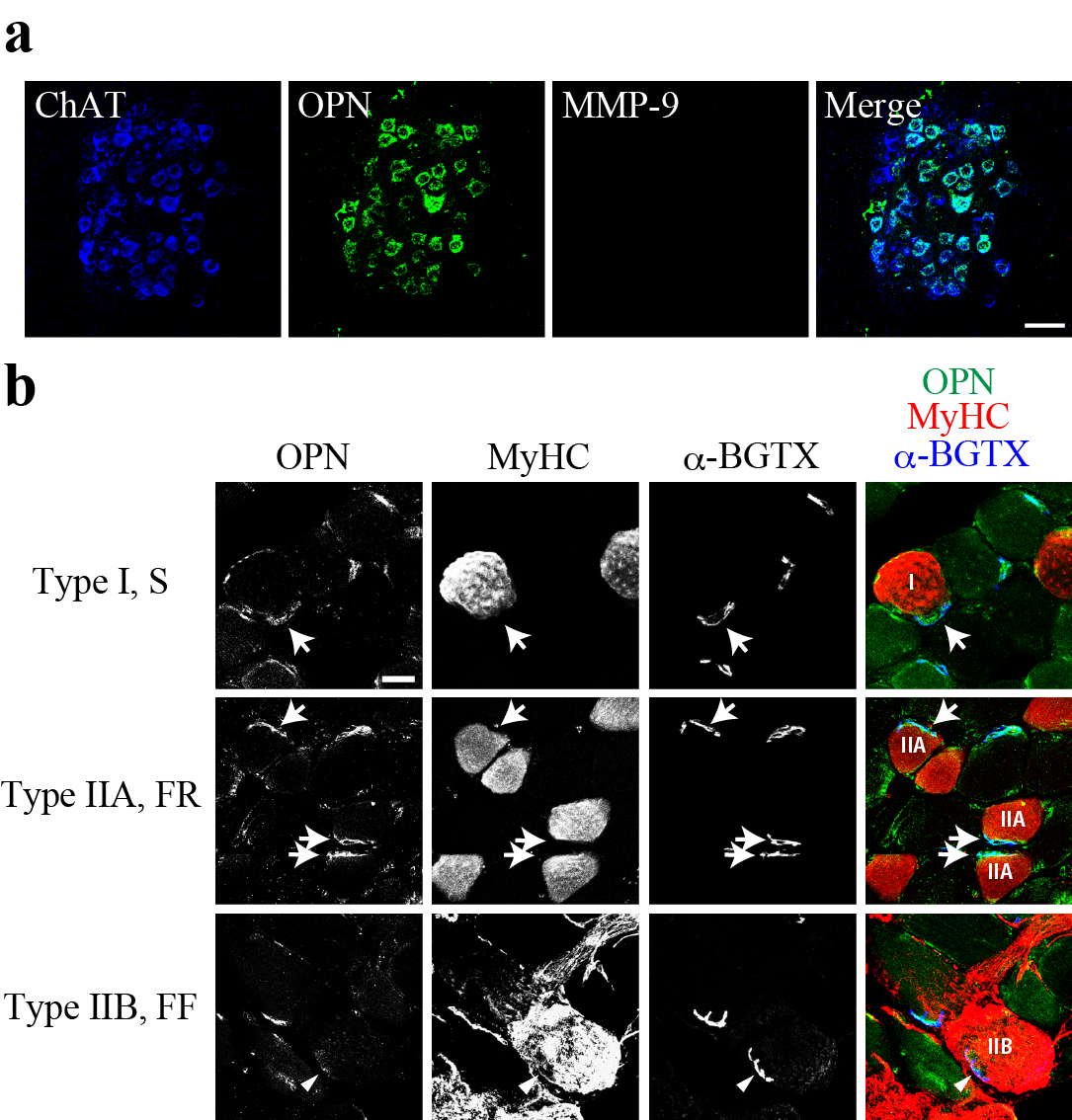
**

**Supplementary Figure 1. OPN expression in the oculomotor nucleus and at neuromuscular junctions on type I and IIA muscle fibers. (a)** Immunostaining for ChAT (blue), OPN (green) and MMP-9 (red) shows negligible MMP-9 expression in the oculomotor nucleus of P100 mice. **(b)** OPN-positive nerve terminals and acetylcholine receptor clusters labeled with α-bungarotoxin (α-BGTX) overlap on Type I and Type IIA muscle fibers (arrows), but not on the Type IIB fibers (arrowhead) in the medial gastrocnemius muscle. Labeling of specific fiber types (I, IIA or IIB) was with appropriate monoclonal anti-myosin heavy chain (MyHC) antibodies. In the type IIB MyHC staining, interstitial tissue is also labeled because of the secondary antibody (anti-mouse IgM; see Experimental Procedures). Scale bar, 100 μm **(a)** and 25 μm **(b)**.


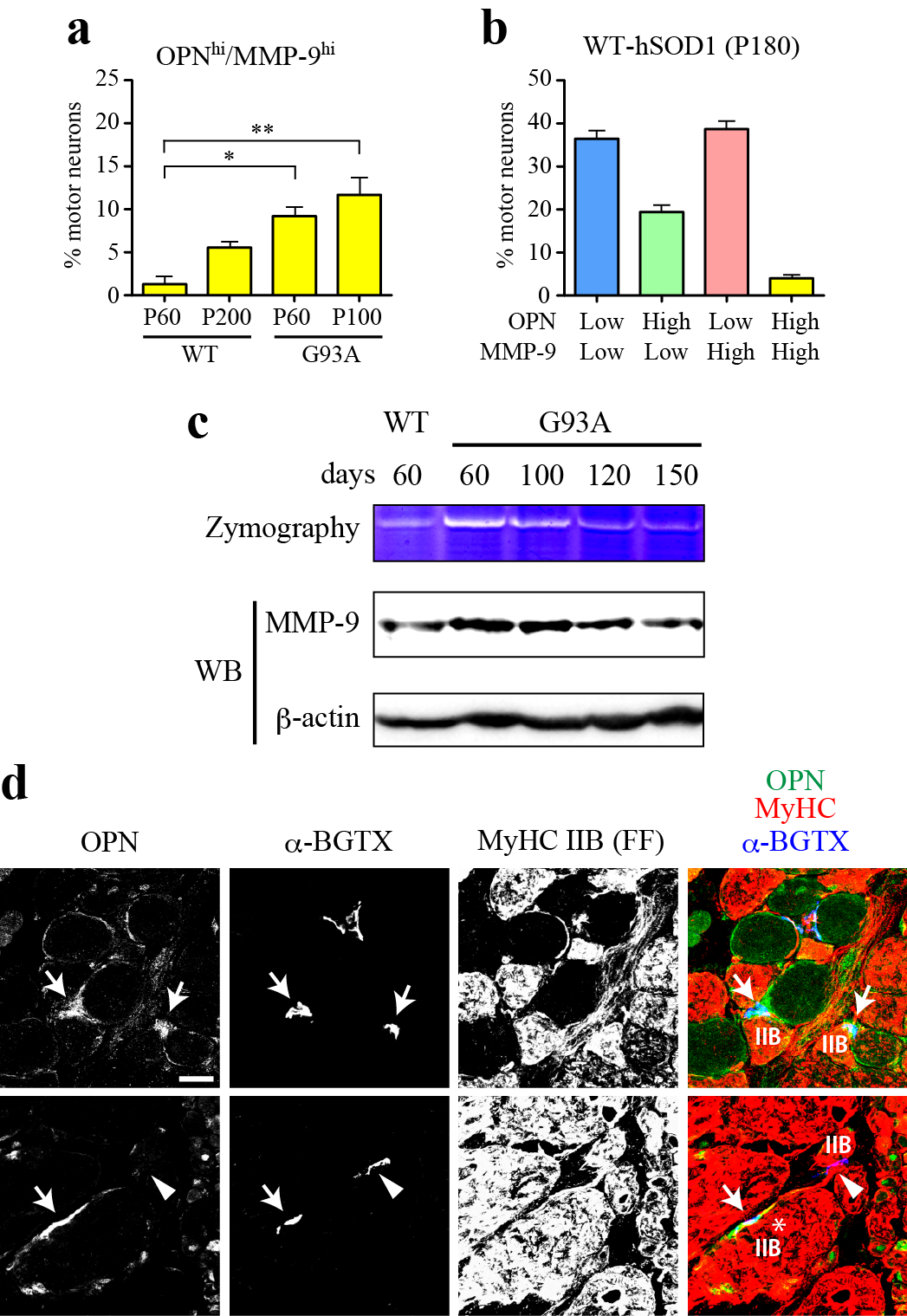


**Supplementary Figure 2. Increased MMP-9 expression in the spinal cord and remodeled OPN-positive MN terminals on Type IIB muscle fibers.**

**(a)** Changes in OPN-high/MMP-9-high MN population (%) among the total surviving MNs in WT and SOD1^G93A^ mice (n=3-4, with 209-595 MNs/8-12 histological sections analyzed per experimental animal; **p*<0.05, ** *p*<0.01, two-tailed Student’s test). (Legend cont’d)

**Supplementary Figure 2.** (cont’d) **(b)** The percentage of MN population classified by OPN/MMP-9 expression in human SOD1(WT) transgenic mice at P180 (n=3, with 405-487 MNs/15 sections analyzed per animal). **(c)** Zymography and Western blot analyses of spinal cord lysates reveal a transient increase in MMP-9 expression on P60 and P100 in SOD1^G93A^ mice. Representative gel and blot out of four experiments. **(d)** In the medial gastrocnemius muscle of P100 SOD1^G93A^ mice, some OPN-positive nerve terminals make contact with muscle nicotinic acetylcholine receptor clusters labeled with α-bungarotoxin (α-BGTX) on type IIB muscle fibers. The region covered by OPN-positive terminals is enlarged on the hypertrophied type IIB fiber (asterisk), but diminished on atrophied fibers (arrowhead). Arrows show OPN-positive terminals on type IIB muscle fibers. Scale bar, 25 μm.


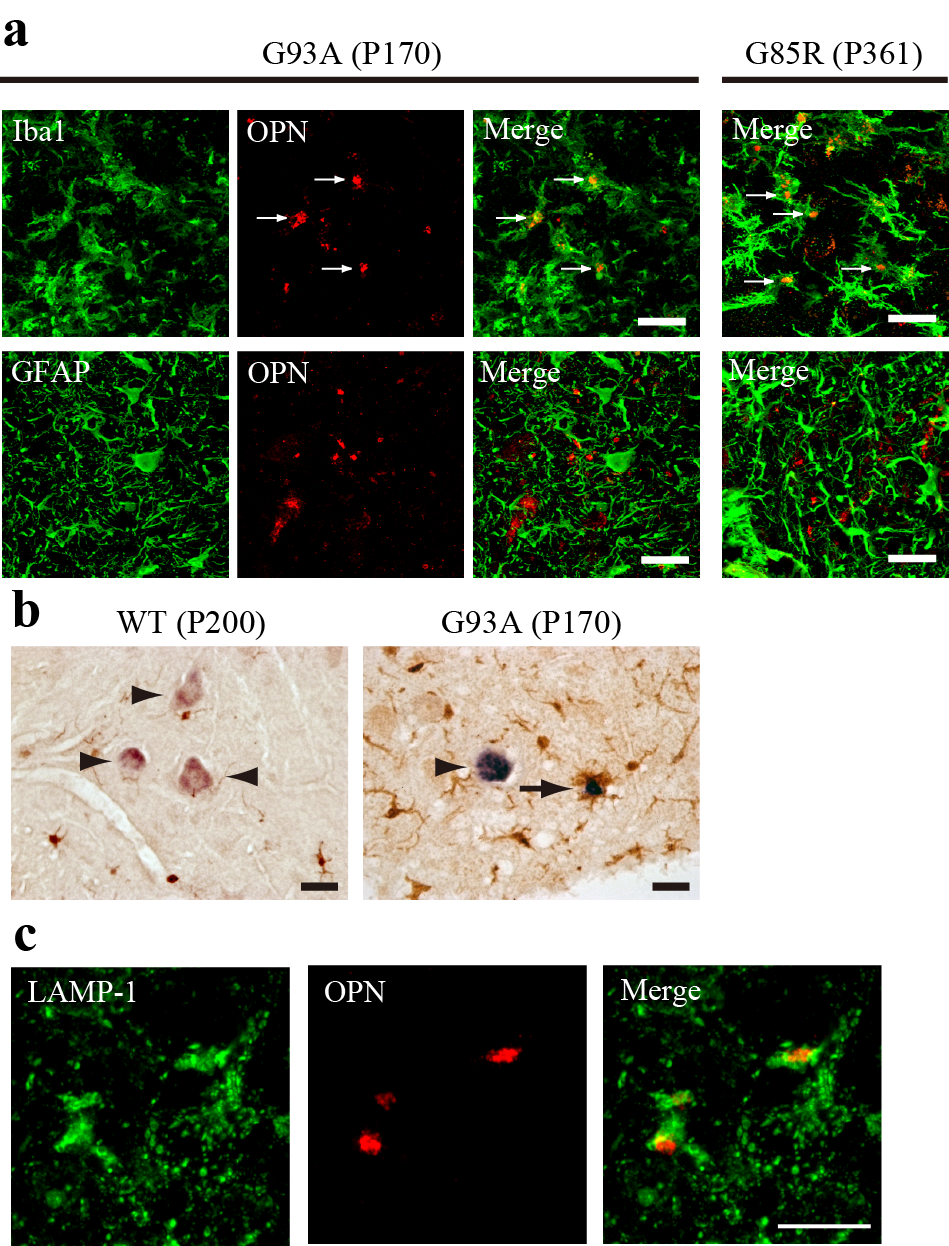
**Supplementary Figure 3. OPN-positive extracellular granules are phagocytosed by microglia/macrophage.** **(a)** Confocal microscopic analysis of OPN-signals and glial markers in the lumbar spinal cords of SOD1^G93A^ (G93A) and SOD1^G85R^ (G85R) mice at their respective end stages. OPN-positive granules were detected within Iba1-positive cells (but not GFAP-positive cells), and are indicated by arrows. **(b)** OPN mRNA was detected using in-situ hybridization (purple) followed by Iba1 immunohistochemistry (brown) in paraffin-embedded sections from WT and G93A mice. Possible OPN-expressing MNs (arrowheads) and OPN-expressing Iba1-positive cells (arrow) are shown. **(c)** Confocal analysis of OPN and LAMP-1 in the lumbar spinal cords of G93A mice (P170). Scale bar, 50 μm **(a, b)**, 10 μm **(c)**.


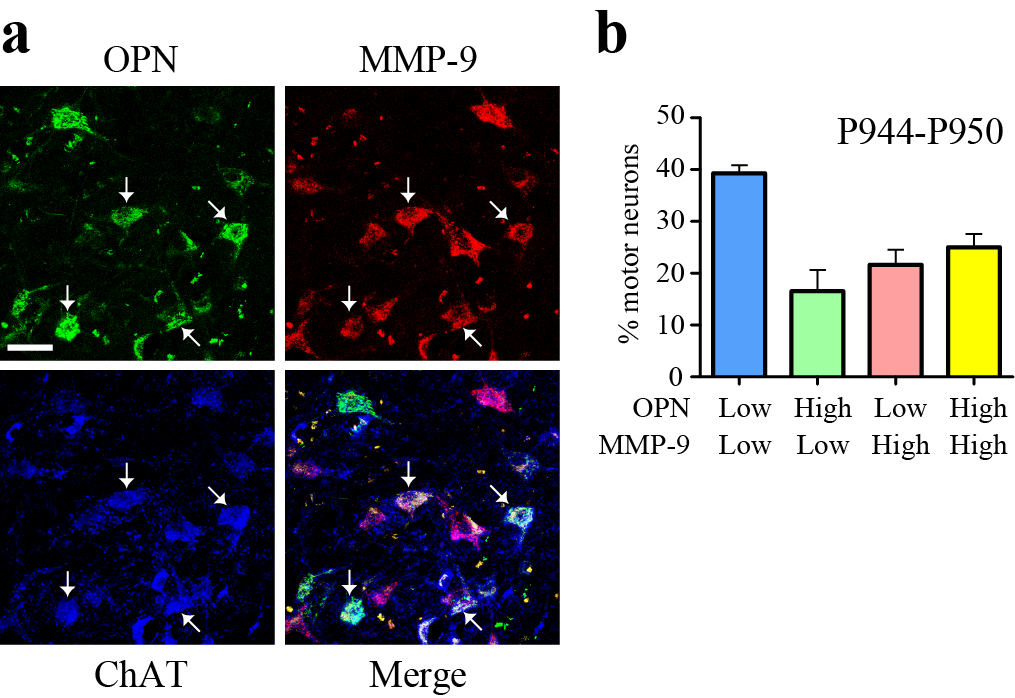


**Supplementary Figure 4. The OPN-high/MMP-9-high MN fraction was increased in aged mice. (a)** Immunostaining for OPN (green), MMP-9 (red) and ChAT (blue) in the lumbar spinal cord of normal aged mice (P950). Arrows indicate OPN^hi^/MMP-9^hi^ MNs. **(b)** MN fractions classified based OPN/MMP-9 expression profiles in the aged mouse spinal cord (n=3; with 147-179 MNs/5-7 histological sections analyzed per experimental animal, P944-P950). Scale bar, 50 μm **(a)**.


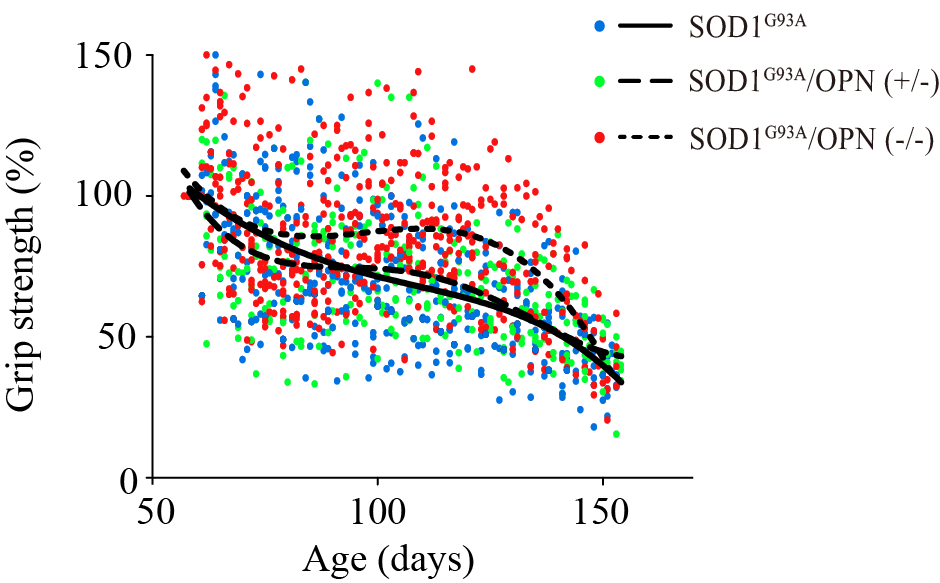


**Supplementary Figure 5. Delayed decline in muscle strength in SOD^G93A^/OPN^−/−^ mice.** Genetic ablation of OPN led to a sustained plateau phase in grip strength decline around the time of disease onset in SOD^G93A^/OPN^–/–^ mice (P100-P120). Grip strength measured on P60 in each genotype (n=14-21 for each) was arbitrarily set to 100%, and the values obtained biweekly were plotted. The fitting curves were drawn with using a four-dimensional polynominal model.


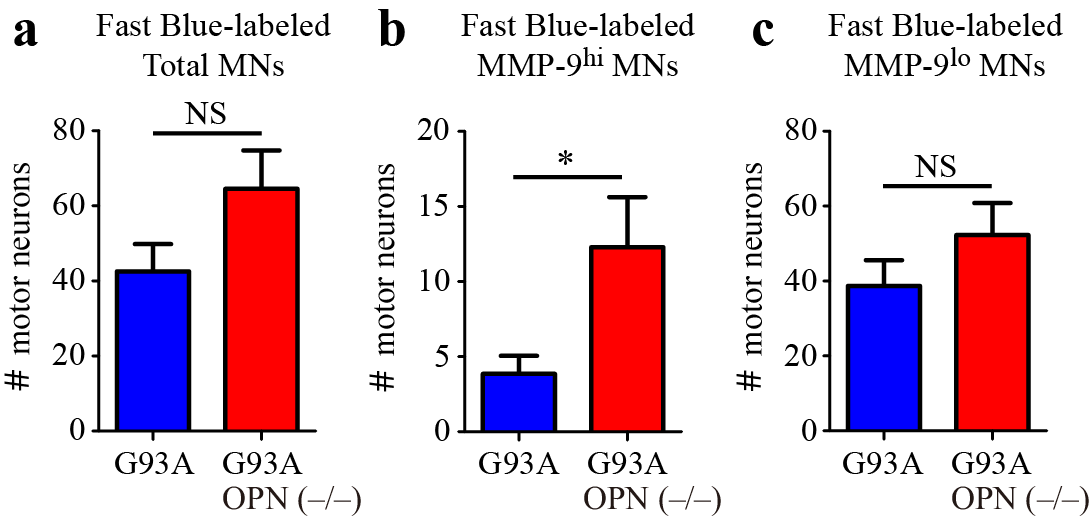


**Supplementary Figure 6. Slowed degeneration of MMP-9-positive remodeled MNs in OPN-deficient SOD1^G93A^ mice as revealed by retrograde MN labeling. (a-c)** Fast blue was injected into the gastrocnemius muscle (P97) of SOD1^G93A^ and SOD1^G93A^/OPN^−/−^ mice (n=7 each). A 10-mm region of the lumbar enlargement of the spinal cord was removed at P100. Serial sections (ca. 200 sections/each spinal cord) were stained with MMP-9 and ChAT. The fast blue-positive retrogradely labeled MNs **(a)** were then classified into MMP-9(-)/ChAT(+) MNs **(b)** or MMP-9(+)/ChAT(+) MNs **(c)**. *p<0.05, two-tailed Student’s test.

**
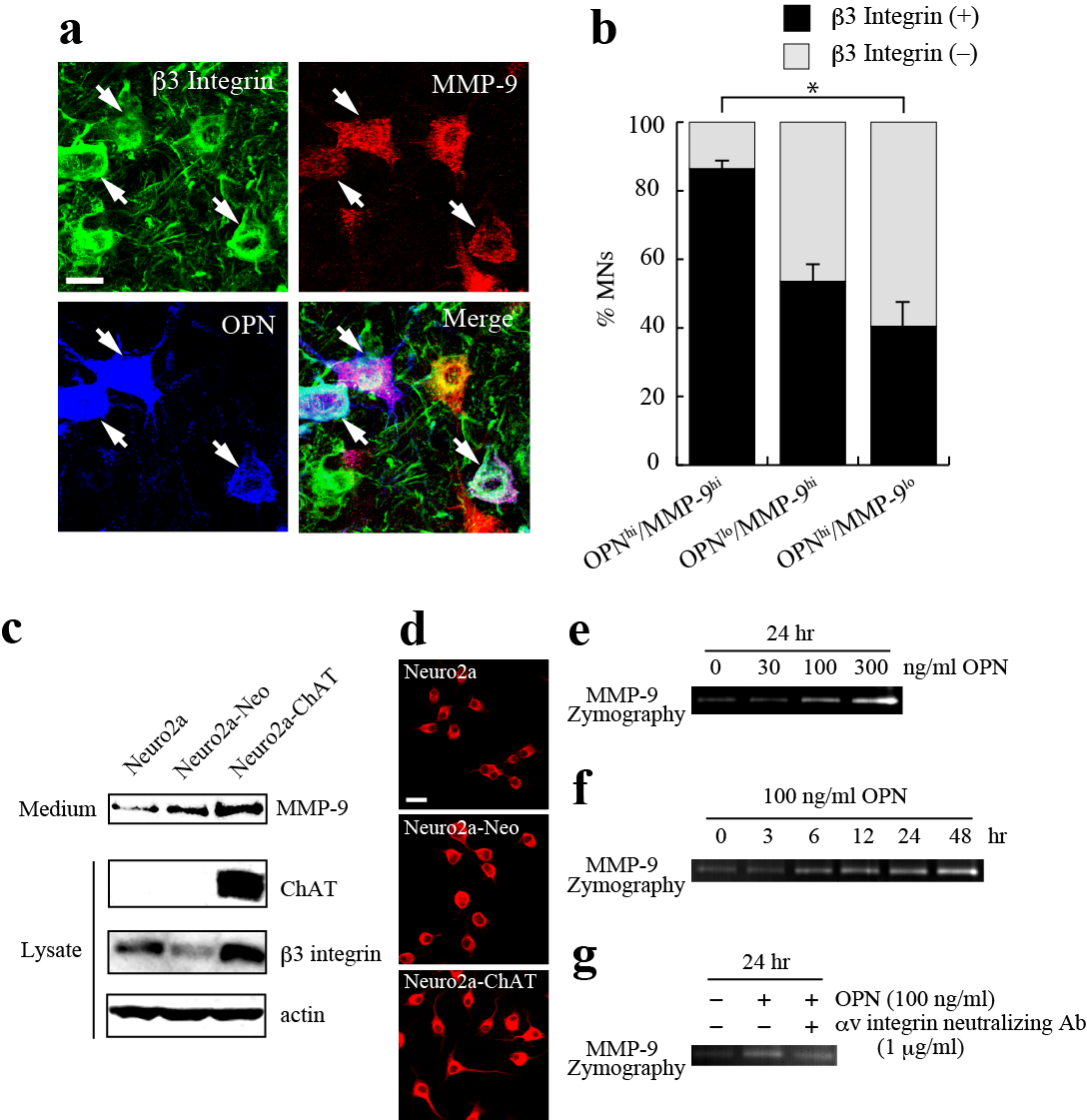
**

**Supplementary Figure 7. Expression of β3 integrin in OPN/MMP-9 double positive MNs in SOD1^G93A^ mice and OPN-induced MMP-9 expression through αvβ3 integrin in Neuro2a-ChAT cells.**

**(a)** Immunostaining for β3 integrin (green), MMP-9 (red) and OPN (blue) in the lumbar spinal cord of SOD1^G93A^ mice on P60. The arrows indicate β3 integrin/MMP-9/OPN triple-positive MNs. **(b)** β3 integrin-positive fraction among MNs classified based their OPN/MMP-9 expression profile in the lumbar spinal cord of SOD1^G93A^ mice on P60. Bars show mean +/- SEM of counts from 3 mice (n = 51-81 MNs for OPN^hi^/MMP-9^hi^, 30-41 MNs for OPN^lo^/MMP-9^hi^, 45-62 MNs for OPN^hi^/MMP-9^lo^, *p<0.05, one-way ANOVA with Tukey-Kramer post hoc tests). (Legend cont’d)

**Supplementary Figure 7. Legend (cont’d) (c)** Western blot analysis of MMP-9 secreted into the medium and ChAT and β3 integrin in cell lysates of parental, mock-transfected or ChAT-transfected Neuro2a cell clones. Representative blot out of two experiments. **(d)** Cell morphology revealed by immunostaining for βIII-tubulin in Neuro2a or stable cell clones. **(e)** MMP-9 zymography showing dose dependency of OPN in the culture medium from Neuro2a-ChAT cells. (**f**) Zymography showing the time-dependent increase in MMP-9 levels in culture medium from Neuro2a-ChAT cells in the presence of 100 ng/ml of OPN. (**g**) MMP-9 zymography showing inhibitory action of an anti-αv integrin neutralizing antibody on OPN-stimulated MMP-9 secretion from Neuro2a-ChAT cells. **(e,f,g)** Representative gel out of three experiments. Scale bar, 25 μm **(a)** and 20 μm **(d)**.


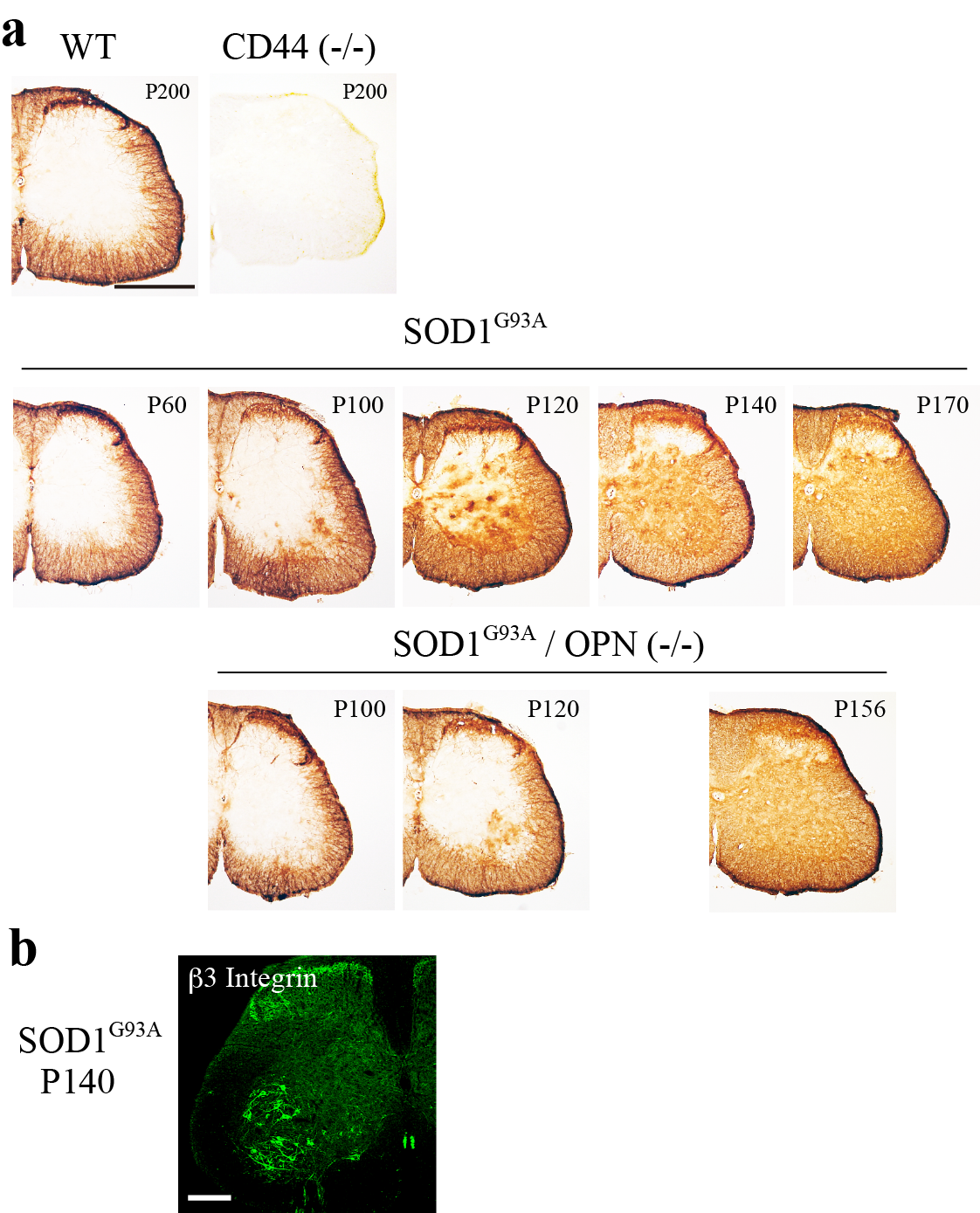


**Supplementary Figure 8. Spinal cord CD44 expression examined using an antibody recognizing different epitope (clone IM7). (a)** Antibody specificity tested using CD44-null mice. Comparison of CD44 expression between SOD1^G93A^ and SOD^G93A^/OPN^−/−^ mice during the disease course. The staining pattern was virtually identical to those in Fig. 6. **(b)** Immunostaining for β3 integrin in spinal cord sections collected from SOD1^G93A^ mice on P140. Scale bar, 500 μm **(a)** and 200 μm **(b)**.


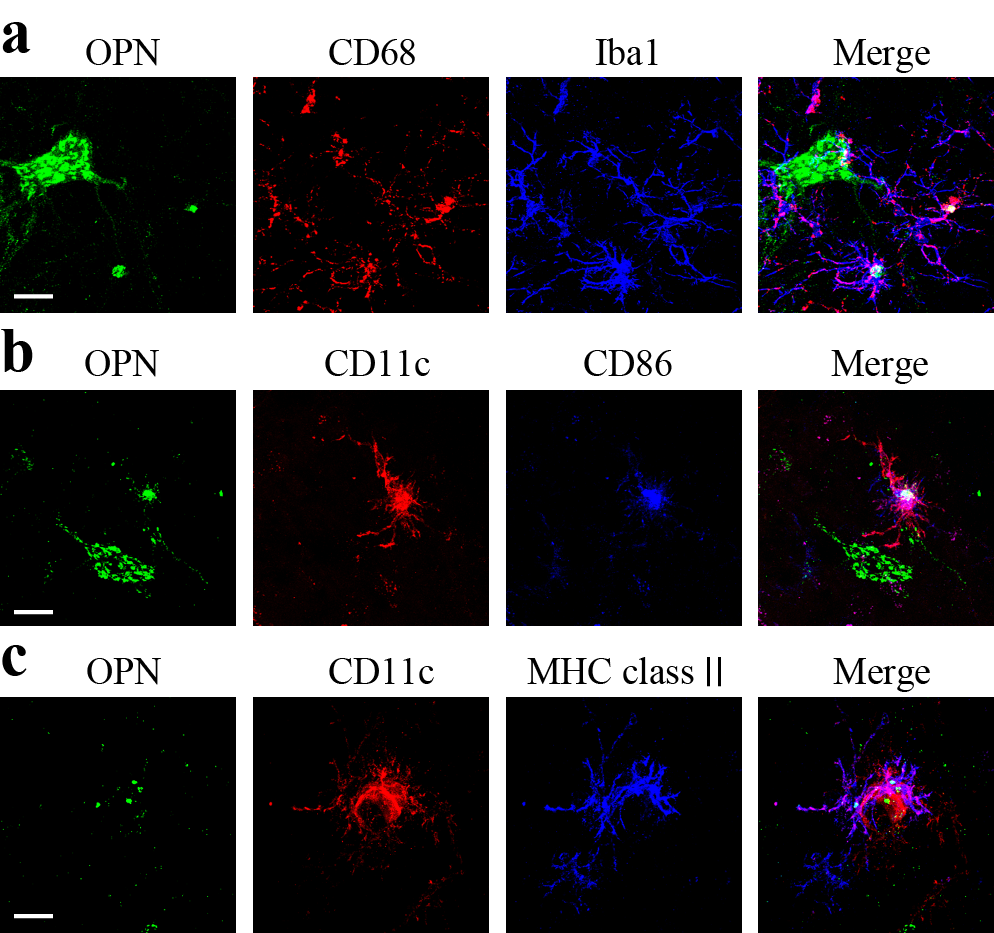


**Supplementary Figure 9. Phenotypic analysis of OPN-phagocytosing microglia/macrophage in the ventral horn of SOD1^G93A^ mice on P100. (a)** Triple immunostaining of OPN (green), CD68 (red) and Iba1 (blue). **(b)** Tripe immunostaining of OPN (green), CD11c (red) and CD86 (blue). **(c)** Tripe immunostaining of OPN (green), CD11c (red) and MHC class II (blue). Scale bar, 20 μm.

**
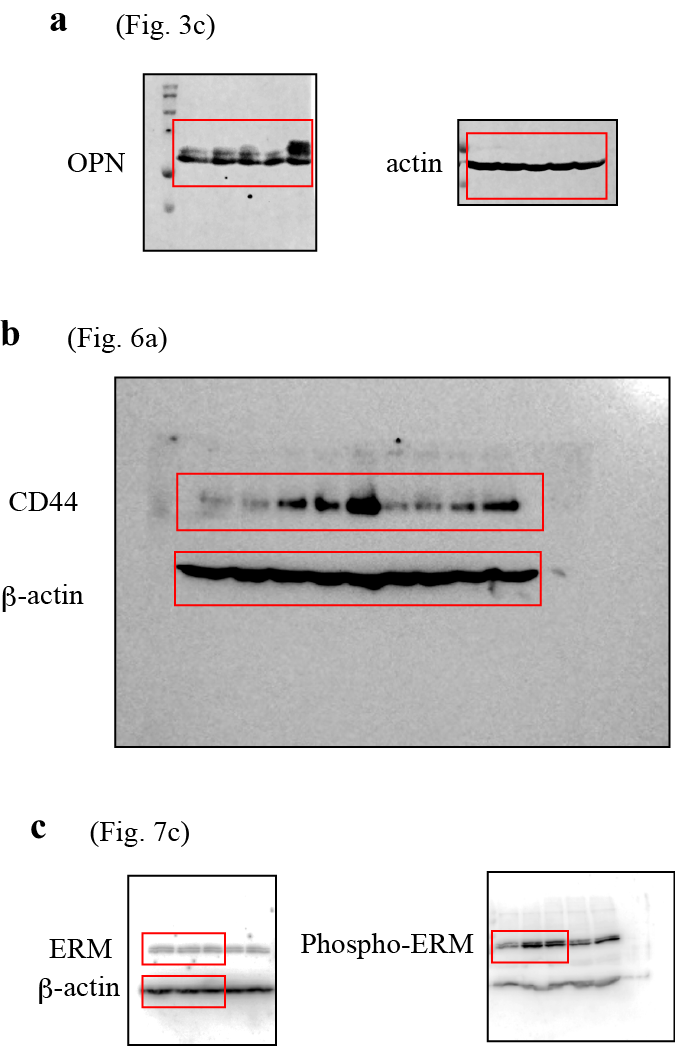
**

**Supplementary Figure 10. Original Western blots of trimmed panels in Fig. 3a, 6a and 7c.**

**Supplementary Table 1. Clinical characteristics of patients used in the study.** The FALS case (case 1) was described in elsewhere in detail (see text). All cases diagnosed with SALS (cases 2-9) were positive for Bunina bodies and neuronal intracytoplasmic Lewy-body-like hyaline inclusions. TDP-43 immunohistochemistry revealed the presence of intracytoplasmic aggregates or diffuse cytoplasmic staining of the remaining motor neurons in all FALS cases.

**Supplementary Table 2. Primer sequences used for genoytyping and qRT-PCR.**
